# *Christensenella minuta* interacts with multiple gut bacteria

**DOI:** 10.3389/fmicb.2024.1301073

**Published:** 2024-02-19

**Authors:** Chang Xu, He Jiang, Li-Juan Feng, Min-Zhi Jiang, Yu-Lin Wang, Shuang-Jiang Liu

**Affiliations:** ^1^State Key Laboratory of Microbial Technology, Shandong University, Qingdao, China; ^2^State Key Laboratory of Microbial Resources, Institute of Microbiology, Chinese Academy of Sciences, Beijing, China

**Keywords:** Christensenellaceae, intestinal microorganism, co-occurrence network, *Faecalibacterium prausnitzii*, *Klebsiella pneumoniae*, nutrient cross-feeding

## Abstract

**Introduction:**

Gut microbes form complex networks that significantly influence host health and disease treatment. Interventions with the probiotic bacteria on the gut microbiota have been demonstrated to improve host well-being. As a representative of next-generation probiotics, *Christensenella minuta* (*C. minuta*) plays a critical role in regulating energy balance and metabolic homeostasis in human bodies, showing potential in treating metabolic disorders and reducing inflammation. However, interactions of *C. minuta* with the members of the networked gut microbiota have rarely been explored.

**Methods:**

In this study, we investigated the impact of *C. minuta* on fecal microbiota via metagenomic sequencing, focusing on retrieving bacterial strains and coculture assays of *C. minuta* with associated microbial partners.

**Results:**

Our results showed that *C. minuta* intervention significantly reduced the diversity of fecal microorganisms, but specifically enhanced some groups of bacteria, such as Lactobacillaceae. *C. minuta* selectively enriched bacterial pathways that compensated for its metabolic defects on vitamin B1, B12, serine, and glutamate synthesis. Meanwhile, *C. minuta* cross-feeds *Faecalibacterium prausnitzii* and other bacteria via the production of arginine, branched-chain amino acids, fumaric acids and short-chain fatty acids (SCFAs), such as acetic. Both metagenomic data analysis and culture experiments revealed that *C. minuta* negatively correlated with *Klebsiella pneumoniae* and 14 other bacterial taxa, while positively correlated with *F. prausnitzii*. Our results advance our comprehension of *C. minuta*’s in modulating the gut microbial network.

**Conclusions:**

*C. minuta* disrupts the composition of the fecal microbiota. This disturbance is manifested through cross-feeding, nutritional competition, and supplementation of its own metabolic deficiencies, resulting in the specific enrichment or inhibition of the growth of certain bacteria. This study will shed light on the application of C. minuta as a probiotic for effective interventions on gut microbiomes and improvement of host health.

## 1 Introduction

The gastrointestinal microbiomes (GMs), as a complex microbial community, are tightly associated with the host health and diseases (Lagier et al., 2018; Dominguez-Bello et al., 2019; de Vos et al., 2022). It is home to hundreds of bacterial species that interact and form networks influenced by diet, physiology, immune response, and probiotics (Hiseni et al., 2021). GMs share the gastrointestinal environment, interact with each other, and associate as a network. Bacterial interactions such as mutualism, commensalism, amensalism or competition (Faust and Raes, 2012) are modulated by food intake, host physiological, and immune systems (Weiss et al., 2023), as well as by probiotics intervention (Ma et al., 2020; Zhang et al., 2023). Due to their complexity (Tierney et al., 2019), it becomes challenging to unravel microbial communities’ dynamics and functions, particularly in terms of the unknown scope of bacterial interactions (Hibbing et al., 2010). Metagenomics is a breakthrough technology that allows comprehensive investigation of the genetic material of microbial communities, helping identify and understand distinct species, their functions, and dynamic changes within the communities upon the intricate interplay between environmental factors, interventions, and diseases (Bäckhed et al., 2015; Chu et al., 2021; Shin et al., 2021; Zhang et al., 2021; Ni et al., 2023). Although data-analytical tools such as co-occurrence networking (Mac Aogáin et al., 2021; Narayana et al., 2023) have been used to depict bacterial relations and interactions, the predictions need to be experimentally validated (Jiang et al., 2022). Such validation has been bottlenecked so far by the shortage of cultivated gut microbial resources and also by fastidious workloads (Chang et al., 2019; Liu C. et al., 2021; Abdugheni et al., 2022). The Christensenellaceae family is a bacterial family that has gained increasing attention due to its important role as gut commensal bacteria in human health (Pascal et al., 2017; Lee-Sarwar et al., 2019; Waters and Ley, 2019; Li et al., 2020; Pittayanon et al., 2020; Prochazkova et al., 2021; Tavella et al., 2021; Villanueva-Millan et al., 2022). Early studies revealed that Christensenellaceae was negatively related to body mass index (BMI) and highly heritable in the human guts (Goodrich et al., 2014; Fu et al., 2015; Ruaud et al., 2020; Mazier et al., 2021). Take *Christensenella minuta* as an example, previous results showed its potential as a probiotic to reduce body weight and inhibit inflammatory reactions (Goodrich et al., 2014; Yang et al., 2018;Mazier et al., 2021; Pan et al., 2022). Thus, *C. minuta* has significant potential for developing treatment of obesity, metabolic disorders, and inflammatory bowel disease (IBD), such as Crohn’s disease and ulcerative colitis (Kropp et al., 2021; Relizani et al., 2022). A mouse gavage experiment with a *C. minuta* strain from healthy human gut in some previous research, showed beneficial effects including reducing blood sugar and blood lipids in high-fat dieted mice (Mazier et al., 2021; Pan et al., 2022). In addition, *C. minuta* produced high levels of acetic acid and moderate levels of butyric acid (Ruaud et al., 2020; Kropp et al., 2021). It has been reported to influence gut microbial diversity and up-regulate the abundance of multiple gut commensal bacteria in previous research, especially for some beneficial bacteria such as *Bifidobacterium* spp. and *Phascolarctobacterium* spp. (Mazier et al., 2021; Pan et al., 2022). For instance, it co-occurred with *Methanobrevibacter smithii* and *Oscillospira* in multiple population investigations (Goodrich et al., 2014; Klimenko et al., 2018; Ruaud et al., 2020). Similar results were also found in animal experiments: providing *C. minuta* to germ-free mice transplanted with *Christensenellaceae*-deficient fecal microbiota from obese humans resulted in the increased abundance of *Oscillospira* (Goodrich et al., 2014). However, the mechanisms by which *C. minuta* interacts with other microorganisms remain unclear with few researches focused on cross-feeding via nutrient metabolisms. Ruaud et al. found that *C. minuta* enhanced the metabolism of *M. smithii* via H_2_ production, which subsequently shifted its fermentation toward acetate instead of butyrate (Ruaud et al., 2020). In addition, *C. minuta* has limitations in serine, cystine and vitamin B12 biosynthesis (Xiao et al., 2019), suggesting the resources of food nutrition or production by other gut microbes are essential for the growth of *C. minuta*. In addition, *Faecalibacterium prausnitzii* and *C. minuta* have been previously noted to have significant associations in terms of their relative abundances (Otten et al., 2021). However, the specific mechanisms by which *C. minuta* interacts with other microorganisms remain unclear.

In this study, we enriched the fecal microbiota with or without *C. minuta* and employed *in vitro* co-culture with metagenomic sequencing techniques to examine the dynamics of the microbial community in response to *C. minuta* intervention, and experimentally validated the interaction of *C. minuta* with predicted bacterial species. The results showed that intervention with *C. minuta* reduced the microbial diversity but selectively enriched the bacterial metabolic pathways that compensated for its metabolic defects. The positive and negative interactions of *C. minuta* with other bacteria were mainly governed from nutrient cross-feeding and competition, respectively. Our results provide clues to understand how *C. minuta* intervention would exert on the gut microbial network and further host health, thus, will shed light on the application of *C. minuta* as a probiotic for effective interventions on gut microbiomes and improvement of host health.

## 2 Materials and methods

### 2.1 Culturing and preparation of *Christensenella minuta* SJ-2

*Christensenella minuta* strain SJ-2 obtained from the human feces in our previous project was used in this study under an anaerobic environment (Liu C. et al., 2021). SJ-2 was recovered from −80°C in glycerol and cultured in modified liquid Gifu Anaerobic Medium (mmGAM) at 37°C (Liu C. et al., 2021). One and a half mL recovered SJ-2 liquid culture was subsequently inoculated in 100 mL mmGAM and cultured until the optical density (OD) 600 value was 1.0 ± 0.05. This SJ-2 suspension was used for fecal sample enrichment in CMe treatment.

### 2.2 Fecal bacterial enrichment and sample collection

Fecal samples were collected from the healthy volunteer who was confirmed to include no detectable amount of *C. minuta* by metagenomic sequencing. A 2 g fecal sample was suspended in sterile 0.01 M phosphate buffer solution (PBS) and passed through 70 μm cell sieves (BIOLOGIX, Irvine, United States) to eliminate large particles. Five mL filtrate was considered as “Feces” treatment, and utilized for bacterial isolation and sequencing. One and a half mL of the filtrate was inoculated into either 100 mL mmGAM (GAMe), or SJ-2 suspension prepared in section 2.1 (CMe) in an anaerobic culture bottle, and incubated at 37°C for 10 days. Five bottles were performed for each treatment as five biological replicates. After 10 days of incubation, 5 mL suspension was collected from each bottle and stored at −80°C until analysis.

### 2.3 Total DNA extraction, sequence processing and analysis

One mL of harvested liquid culture or fecal slurry was centrifuged at 13,200 × *g* for 5 min, and the pellets were resuspended in sterile 800 μL 0.01 M PBS. Total genomic DNA extraction was performed using the DNeasy PowerSoil Kit following the manufacturer’s instructions (Qiagen, Hilden, Germany). A total of 11 metagenomic libraries (including 1 Feace: fecal seed liquid library, 5 GAMe libraries and 5 CMe libraries) were sequenced by Novogene (Tianjin, China) using an Illumina sequencing platform with NovaSeq (PE150, Santa Clara, CA, United States). Each sample was prepared to obtain libraries with a mean insert size of approximately 350 bp. Raw reads were filtered using SOAPnuke v2.1.7 (Chen et al., 2018), and first aligned with the human genome [alignment with *Homo sapiens* (human)] (RefSeq GCF_000001405.39). The reads aligned to the human genome were removed via bowtie2 v2.3.5.1 (Langmead et al., 2009), and the remaining high-qualified reads in FASTQ format were used for further analysis according to default parameters.

Assembled sequences were combined using Metawrap v1.3.2 (Uritskiy et al., 2018) which also is the ensemble approach binning tool, and individually subsequent binning was achieved using MaxBin2 (Wu et al., 2016), MetaBAT2 (Kang et al., 2019), and CONCOCT (Alneberg et al., 2014) in Metawrap v1.3.2 (Uritskiy et al., 2018). By using MetaWRAP v1.3.2 bin_refinement module (*−c 50* and *−x 10*) (Uritskiy et al., 2018) consolidated and optimized the bin sets obtained by each box splitting tool. Only metagenomic assembled genomes (MAGs) with medium to high quality (completeness ≥50% and contamination ≤10%) were retained for downstream analysis (Bowers et al., 2017). Computational work on the relative abundances of MAGs in different samples was implemented by coverM v0.6.1^1^ with the option *-min-read-percent-identity 0.9* and *-min-read-aligned-percent 0.7* for which these stringent identity and alignment cutoffs were implemented to minimize spurious mappings that may artificially inflate the MAG abundances. The classification was carried out using the classified workflow of GTDB-Tk v1.5.1 (Chaumeil et al., 2022), and a phylogenetic tree based on the whole genomes was constructed using GTDB-Tk v1.5.1 (Chaumeil et al., 2022) with the genomes of *Pseudomonas aeruginosa* (p_Proteobacteria, GCF_000006765.1), which are almost absent in the human gut, used as the outgroup due to its distant taxonomic position.

EnrichM v0.6.4^2^ was used to explore the metabolic potential for the MAGs based on Kyoto Encyclopedia of Genes and Genome (KEGG) Orthogroups (KOs) (Kanehisa and Goto, 2000). The non-redundant gene set was constructed using 91 MAGs with software CD-HIT v4.5.8 (Li and Godzik, 2006), resulting in a total of 205,034 non-redundant genes. Subsequently, EnrichM v0.6.4 was employed for annotation, and the differences in community functionality between groups were compared by analyzing gene coverage. The metabolic potential of MAGs was evaluated by annotating modules using EnrichM v0.6.4. MAGs are considered to have complete metabolic capabilities only when the integrity score of Modules is ≥70% (Botté et al., 2023).

### 2.4 Bacterial co-occurrence network analysis

The coverage and relative abundance of all MAGs in each sample were analyzed using coverM v0.6.1 (see footnote 1). Nine total samples were assessed for correlation relatedness as continuous change points using eLsa v1.0.2 (Xia et al., 2011) analysis tools. The computational correlation results were filtered according to Spearman’s correlation, and the co-occurrence network was visualized by Cytoscape v3.9.1 (Shannon et al., 2003).

### 2.5 Isolation and identification of gut bacterial species

One mL of fecal filtrate was serially diluted using 0.01 M PBS and 100 μL of the dilution within specified ranges was spread on 150 cm^2^ agar plates containing anoxic medium, which were then incubated at 37°C for 21 days. Sixteen different culture conditions were used (Supplementary Table S1). Single colonies appearing on the plates were picked, with 25–30 colonies selected from each plate on the 3rd, 10th, and 21st day, totalling 3 times, and re-streaked onto corresponding agar for purity. The identification of bacterial isolates was achieved via full-length 16S rRNA gene sequencing. Polymerase chain reaction amplicons of the 16S rRNA gene utilizing universal primers 27F (5′-AGAGTTTGATCCTGGCTCAG-3′) and 1492R (5′-GGTTACCTTGTTACGACTT-3′) were sent for Sanger sequencing by Sangon (Qingdao, China) and subsequently identified via EzBioCloud^4^ (Yoon et al., 2017) and NCBI (National Center for Biotechnology Information^5^) blast (Federhen, 2012). We used the Neighbor-Joining algorithm with 1,000 bootstrap replicates to construct the phylogenetic evolutionary tree. The multiple sequence alignment of the 16S rRNA gene sequences of 121 isolated strains and one distantly related type strain, *Pseudomonas aeruginosa* DSM 50071 (NR_026078.1) was performed via the ClustalW function in MEGA11 (Tamura et al., 2021).

### 2.6 *In vitro* interaction assays

The interactions between the bacterial isolates and SJ-2 were validated using plate confrontation and liquid-culture experiments, respectively, (de Vos et al., 2017; Liu P. et al., 2021). To perform the plate confrontation experiment, 2.5 μL of SJ-2 culture at logarithmic phase was vertically dripped onto a mmGAM agar plate, followed by adding 2.5 μL of another bacterium externally tangentially to the circular droplet. In the experimental setup, both SJ-2 and the target bacterium were subjected to an ecological niche preemption treatment, where each of them was given the opportunity to occupy the niche first (represented as the initial point). We carried out the experiment of dropping the potential interactive bacteria culture vertically first, then dropping SJ-2 at the edge, to verify the influence of one side occupying the ecological niche first on the other side, and the two groups of experiments obtained the same results. The plates were cultivated at 37°C for 2–3 days and photographed to record. Observation and documentation of colony morphology were performed under a stereomicroscope (Nikon corporation SMZ18, Tokyo, Japan) with an eyepiece magnification of 10× and a zoom setting of 0.75×. For the liquid-culture experiment, SJ-2 cultured in broth medium was centrifuged at 10,000 × *g* for 15 min after 2 days incubation. The supernatant was filtered using a 0.22 μm filter to remove SJ-2 cells. The conditioned media were prepared by mixing the supernatant with fresh mmGAM medium at equal volume. The tested bacteria were inoculated into the conditioned media at a ratio of 1%. Fresh mmGAM was used to incubate the tested bacteria as a control. The growth rate was determined by measuring the OD at 600 nm under the two conditions during 24 h after inoculation. Due to poor liquid culture conditions and low turbidity of bacterial strains, some bacteria were not subjected to liquid cultivation. Therefore, we performed centrifugal concentration of the bacterial suspension for solid agar-based confrontation experiments.

### 2.7 Mechanism exploration experiment

The physiological and biochemical features of the tested bacteria, including SJ-2 were profiled using ANI MicroPlates (BIOLOG, Hayward, United States) following the manufacturer’s instructions. A volume of 3 μL of fresh *F. prausnitzii* bacterial suspension was inoculated into each well of a 96-well plate (NEST, Tianjin, China) containing 150 μL of preheated culture medium. The plate was then incubated for 24–36 h at 37°C with >95% humidity in a growth curve analyzer (BMG LABTECH SPECTROstar Omega, BMG LABTECH, Ortenberg, Germany) under static incubation conditions and anaerobic environment. The OD 600 was measured approximately every 30 min using a plate reader. Prior to each measurement, the growth curve analyzer was shaken at 700 rpm for 5 min. The growth status of each well culture over time was determined by monitoring the increase in OD 600 (reducing the initial value at 0 h).

Calculation formula for generation time (h). $G=\frac{T_{2}-T_{1}}{\log_{2}\left(\frac{{OD}_{2}}{{OD}_{1}}\right)\text{.}}$

Calculation formula for growth rate (/h). $v=\frac{\ln\left(\frac{{OD}_{2}}{{OD}_{1}}\right)}{T_{2}-T_{1}}$

### 2.8 Statistical analysis

The alpha diversity was calculated using the Shannon diversity index, and its significance was assessed by the Mann–Whitney U test. Principal Coordinates Analysis (PCoA) was selected for the analysis of species composition diversity. The Bray-Curtis Index was chosen as the distance method, and PERMANOVA was selected as the statistical method. The intergroup differences in the relative abundance of MAGs, pathways and modules were analyzed for significance using the Linear discriminant analysis Effect Size (Lefse) test, with a significance threshold set at Linear Discriminant Analysis (LDA) ≥ 2.0. β Diversity was used to explore differences between pathway abundance in samples using Principal component analysis (PCA). The potentially interacted bacteria were selected based on the Spearman correlation coefficient between their abundance and the changes in SJ-2 abundance (correlation: SCC ≥ 0.6 or ≤ −0.6). In order to detect changes in the gut microbial ecosystem caused by SJ-2, we removed all the reads assigned as SJ-2 from the dataset and re-normalized them when analyzing community structure and function (Mazier et al., 2021). Mean and standard error of the mean (SEM) were used to analyze and visualize the data. All statistical analysis was performed using IBM^®^ SPSS^®^ Statistics 23, including tests for normal distribution and homogeneity of variance. Depending on the data distribution, *T*-tests, Mann–Whitney’s U test, or Kruskal-Wallis test was used to compare the differences and assess the impact of SJ-2 fermentation broth. The effects of substance addiction on *F. prausnitzii* growth curve were evaluated using repeated one-way ANOVA. To account for multiple testing, *p*-values were corrected using the Benjamini-Hochberg (BH) method (In all the significance tests for differences, *p* ≤ 0.05 is considered significant).

The drawing of stack and Venn diagrams is implemented by R v3.6.1, heat map, drawn by Lefse using imageGP (Chen et al., 2022). To modify the phylogenetic tree, iTOL v5 (Letunic and Bork, 2021) was used. Data processing and growth curve plotting were conducted using GraphPad Prism 9. The co-occurrence network was visualized using Cytoscape v3.9.1 (Shannon et al., 2003).

## 3 Results

### 3.1 Metagenomic sequencing and assembly of gut bacteria

**Figure 1:**
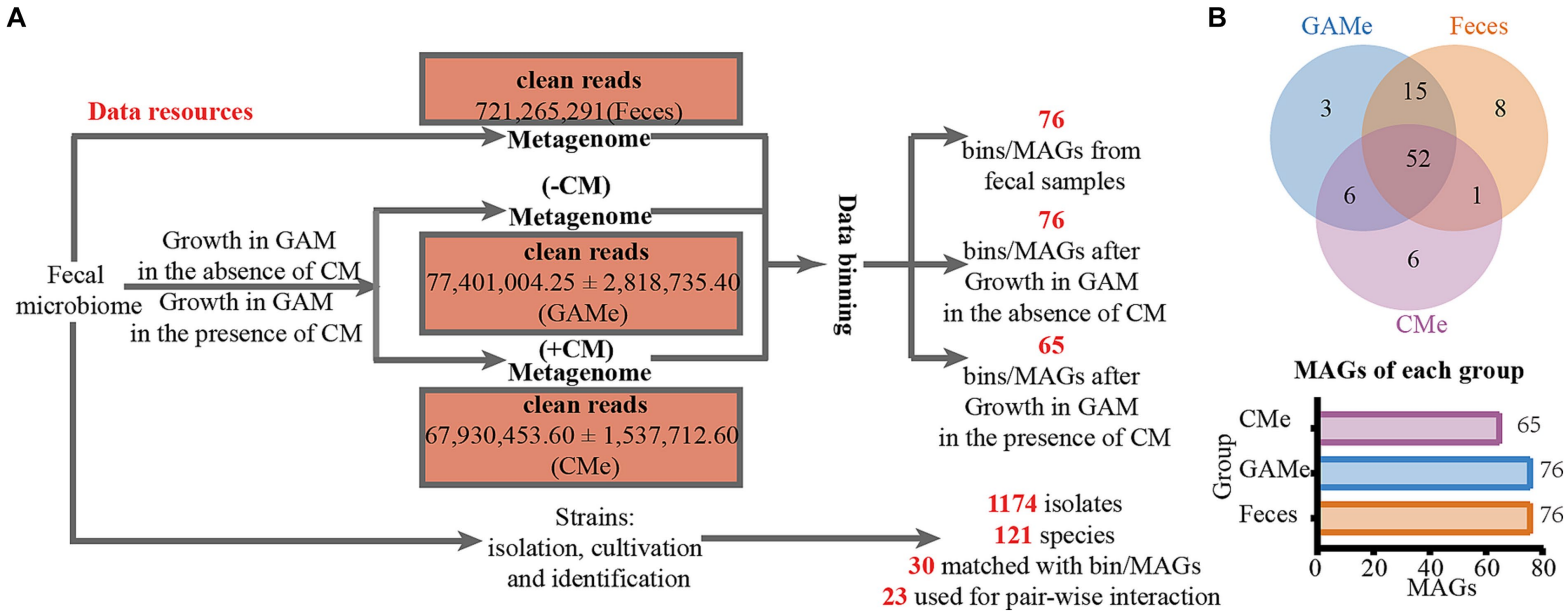
DNA sequencing data obtained in this study. **(A)** Experimental design for resource collections and numbers of metadata and isolates of each treatment. **(B)** Binning of metagenomic data and assembled MAGs from each sample.

Fecal microbiota was cultivated with mmGAM medium in the presence (labeled as CMe) and absence (labeled as GAMe) of *C. minuta* SJ-2. The metagenomes from CMe and GAMe, as well as the original seeding fecal samples (labeled as Feces) were extracted and sequenced. Among the total of 11 genomic libraries, 10 were successfully sequenced with one GAMe sample lost. After quality control and filtration, a total of 721,265,291 clean reads were obtained with averages of 72,009,006 (Feces), 77401004.25 ± 2818735.40 (GAMe), and 67930453.60 ± 1537712.60 (CMe) (Figure 1A, and for the details see Supplementary Table S2). A total of 91 Metagenome-Assembled Genomes (MAGs) were obtained (Supplementary Tables S3, S4), following the criterion of Bowers’ classification standard for intermediate to high quality genomes (Bowers et al., 2017). Of the 91 MAGs, 76 were detected in Feces sample, 76 in the GAMe and 65 in the CMe group (Figure 1B, Supplementary Table S3). 52 MAGs were shared in all three groups, while 8, 3, and 6 were specifically annotated in Feces, GAMe, and CMe groups, respectively (Figure 1B). In addition, the overlapped MAGs of paired groups were 67, 53, and 58 in Feces-GAMe, Feces-CMe, and CMe-GAMe, respectively. The CMe group exhibits a lower number of MAGs compared to the other two groups.

### 3.2 *Christensenella minuta* intervention altered the biodiversity and functionality of fecal microbiomes

**Figure 2:**
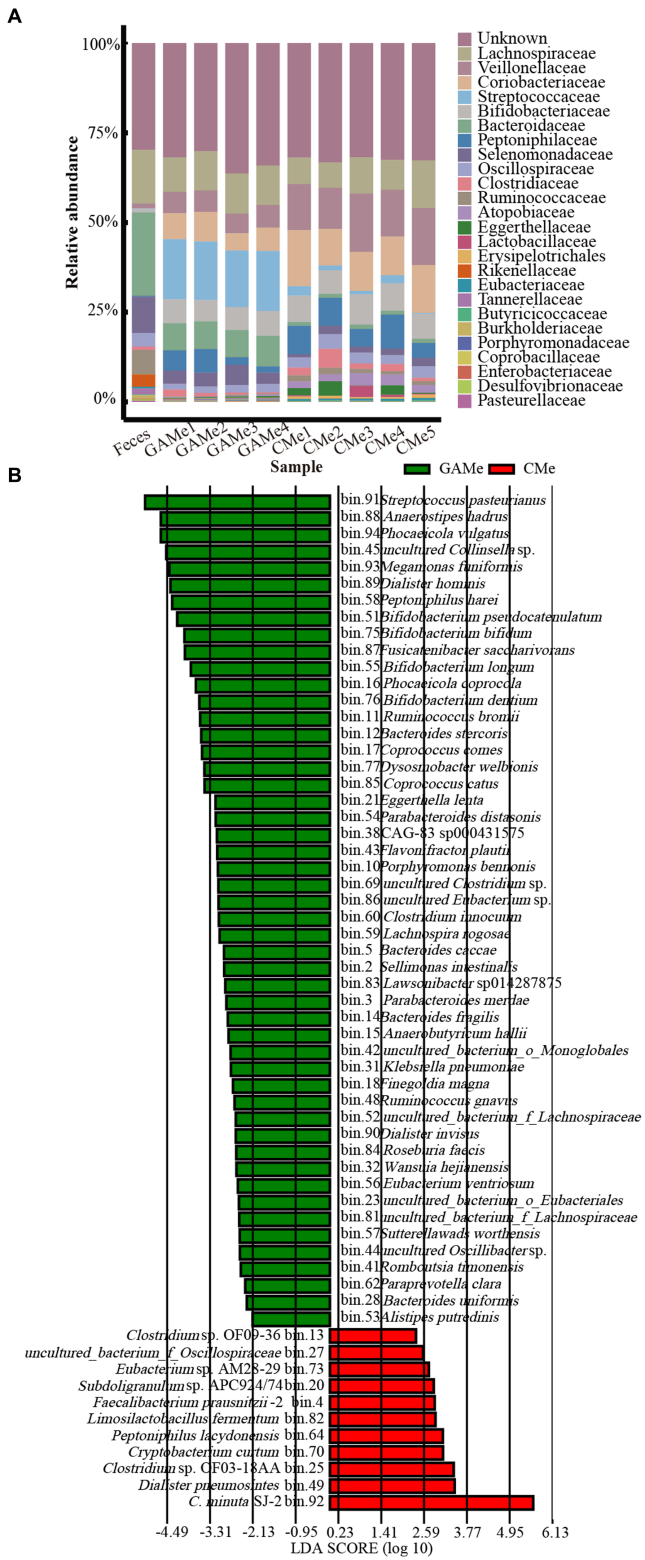
Impacts of *C. minuta* SJ-2 on the species composition of the fecal microbiomes. **(A)** The top 20 abundant taxa at the family level (annotated for MAGs) in fecal, GAMe, and CMe samples. Sequences for Christensenellaceae were removed for this analysis. **(B)** The Linear discriminant analysis Effect size (LefSe) distribution of bacterial taxa between the GAMe group and CMe group. The annotated bacterial species names were shown beside the bin numbers.

Both the alpha and beta diversity analyses showed that there was a notable difference between the enriched community and the fecal microbiota. It is not surprising that the growth in mmGAM resulted in changes in fecal microbial diversity, as demonstrated by a decrease in the Shannon index in both the enriched treatments. Additionally, the intervention of SJ-2 further differentiated the fecal microbiomes, with the alpha diversity of the CMe community being significantly lower than that of the GAMe (Supplementary Figure S1). At the family level, Lachnospiraceae was dominant in all 3 groups, while the relative abundance of other families showed apparent changes. The relative abundance of Veillonellaceae, Coriobacteriaceae, and Bifidobacteriaceae increased consistently in both GAMe and CMe groups, while Bacteroidaceae, Selenomonadaceae, and Rikenellaceae decreased in both groups. Meanwhile, Ruminococcaceae and Oscillospiraceae decreased in GAMe but significantly increased in CMe, while Streptococcaceae increased in GAMe but decreased in CMe. Additionally, Clostridiaceae, Atopobiaceae, Lactobacillaceae, Erysipelotrichaceae, and Eubacteriaceae were specifically enriched in the CMe group (Figure 2A). The LefSe results showed that 50 MAGs were specifically enriched in the GAMe samples, while 10 MAGs (excluding SJ-2) specifically represented a high-level redundancy in the CMe group (LDA ≥ 2). Among those MAGs with significant changes, *Dialister pneumosintes* and *Clostridium* sp. OF03-18AA were the most representative in the CMe, while *Streptococcus pasteurianus*, *Anaerostipes hadrus* and *Phocaeicola vulgatus* were the most abundant in the GAMe (Figure 2B).

**Figure 3:**
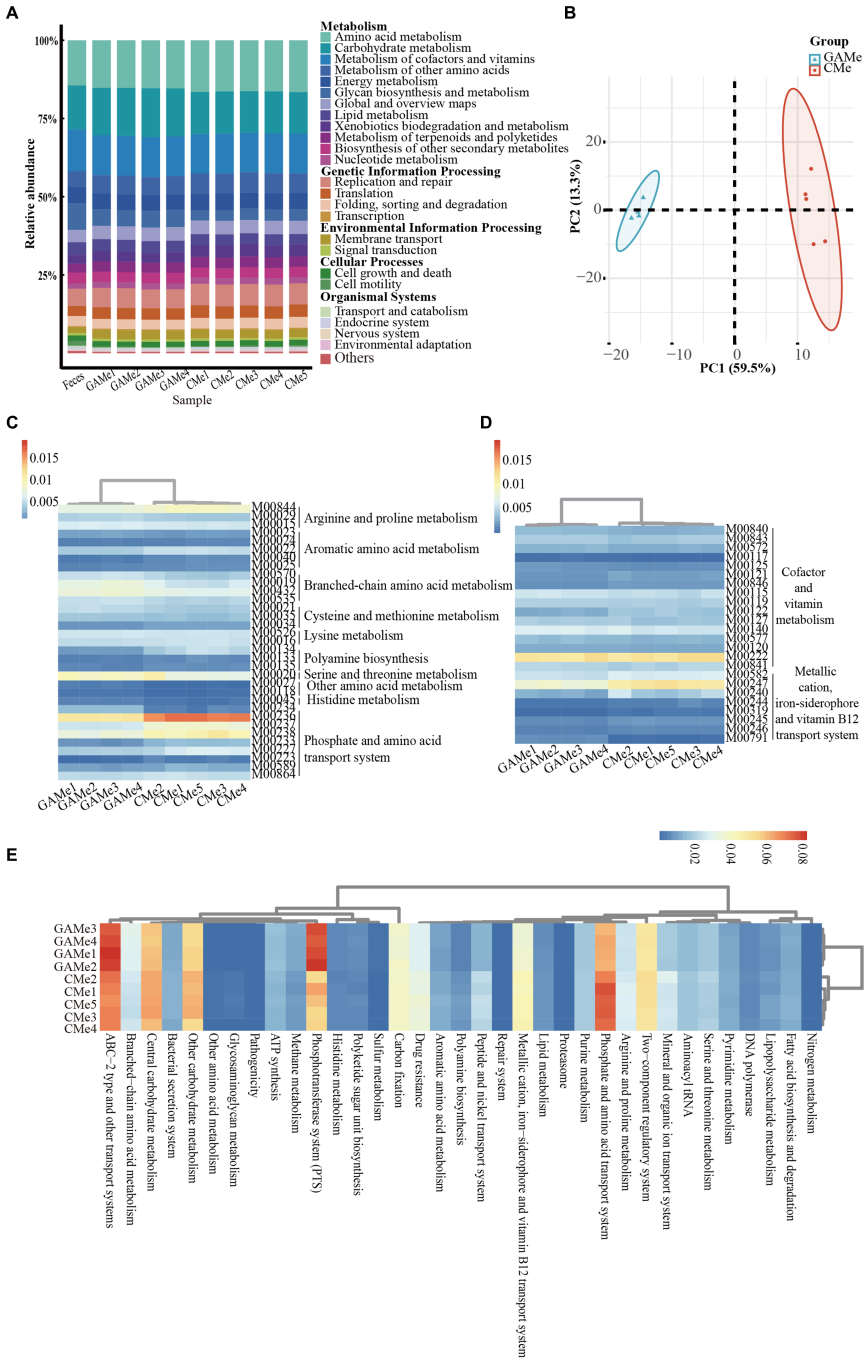
Impacts of *C. minuta* SJ-2 on the functionality of fecal microbiomes. **(A)** Relative abundance of L2 metabolic pathways in three groups. **(B)** Principle component analysis (PCA) score plot comparing the relative abundance of L3 metabolic pathways between GAMe and CMe. **(C)** The heatmap of relative abundance composed of the modules of amino acid metabolism with significant differences (Linear discriminant analysis, LDA ≥ 2) between the GAMe and CMe groups. **(D)** The heatmap of relative abundance composed of the modules of vitamin metabolism with significant differences (LDA ≥ 2) between the GAMe and CMe groups. **(E)** The heatmap of relative abundance composed of L3-level pathways with significant differences (LDA ≥ 2) between the GAMe and CMe groups. The color bars from red to blue indicated the relative abundance from high to low.

The EnrichM software (Fan et al., 2023) was employed to predict the functional capacity of the fecal microbiota based on the metagenome data (Supplementary Table S5). To gain insights into the KEGG pathway features and differences in the three groups, we performed a functional distribution analysis the results were plotted (Figure 3). Despite the changes in the microbial diversity shown above (Supplementary Figure S1), the overall functionality at pathway level 2 of Feces, GAMe and CMe groups were apparently similar (Figure 3A, Supplementary Table S6). Substance transport, sugar and amino acid metabolism, cofactor and vitamin metabolism, drug resistance, as well as fatty acid synthesis and metabolism were highly enriched in all three groups showing their critical roles during gut microbial survival. These features might suggest that the fecal microbiota possessed functional robustness that enables it to accommodate perturbations in environments.

We further explored metabolic modules in the GAMe and CMe groups. A total of 515 metabolic modules were identified in the GAMe and CMe groups, among which 72 significantly enriched modules were contributed by SJ-2 (Supplementary Table S7). In order to identify the true impact of *C. minuta* on the fecal microbiome functionalities, the sequences of *C. minuta* were excluded for further analyses. Performing principal component analysis (PCA) revealed significant differences between the GAMe and CMe groups at the KEGG pathway level 3 (Figure 3B). In general, there were 120 and 101 modules enriched in the GAMe and CMe groups, respectively (Supplementary Table S8). Both the GAMe and CMe groups were abundant in high levels (relative abundances >0.04) of two-component systems, phosphate and amino acid transport systems, phosphotransferase system (PTS), central and other carbohydrate metabolism, ABC-2 type and other transport systems (Supplementary Table S9). The GAMe microbiota particularly enriched PTS and ABC-2 type and other transport systems (Figure 3E, Supplementary Table S9). In the CMe group, the abundances of glutamate, branched-chain amino acids and polar amino acid transport (M00233, M00227, M00223, M00236, M00237), aromatic amino acids (M00040, M00025), and polar amino acid synthesis (M00020) were increased, while polyamine metabolism (M00134, M00133) was enhanced (Figure 3C). Meanwhile, the synthesis of branched-chain amino acids, sulfur-containing amino acids and tryptophan (M00019, M00432, M00570, M00535; M00021, M00035; M00023) showed a significant decrease under SJ-2 intervention (Figure 3C). In the pathways of cofactor and vitamin metabolism, the VB12, VB1, and biotin synthesis modules in the fecal microbiota were significantly enriched under SJ-2 intervention (M00122, M00127, M00577) (Figure 3D). We also observed the enrichment of metabolic pathways such as coenzyme A (M00120), β-oxidation (M00086), and ketone body synthesis (M00088), which regulate the microbiota to a relatively favorable environment (Supplementary Table S10). It seemed that intervention with SJ-2 drove the fecal microbiomes toward the active synthesis of substances that would compensate for SJ-2 metabolic deficiencies such as the synthesis of serine, glutamic acid, tyrosine, biotin, VB12, VB1, thereby enhancing the efficiency of their synthesis in the community after the intervention. On the other hand, the complete metabolic pathways of SJ-2 promoted the enrichment of microorganisms using arginine, branched-chain amino acids, methionine and tryptophan as metabolic substrates, which were directly supplied through SJ-2 to improve material utilization efficiency (Supplementary Table S11).

### 3.3 Co-occurrence network predicts *Christensenella minuta* interacts with multiple bacterial species

**Figure 4:**
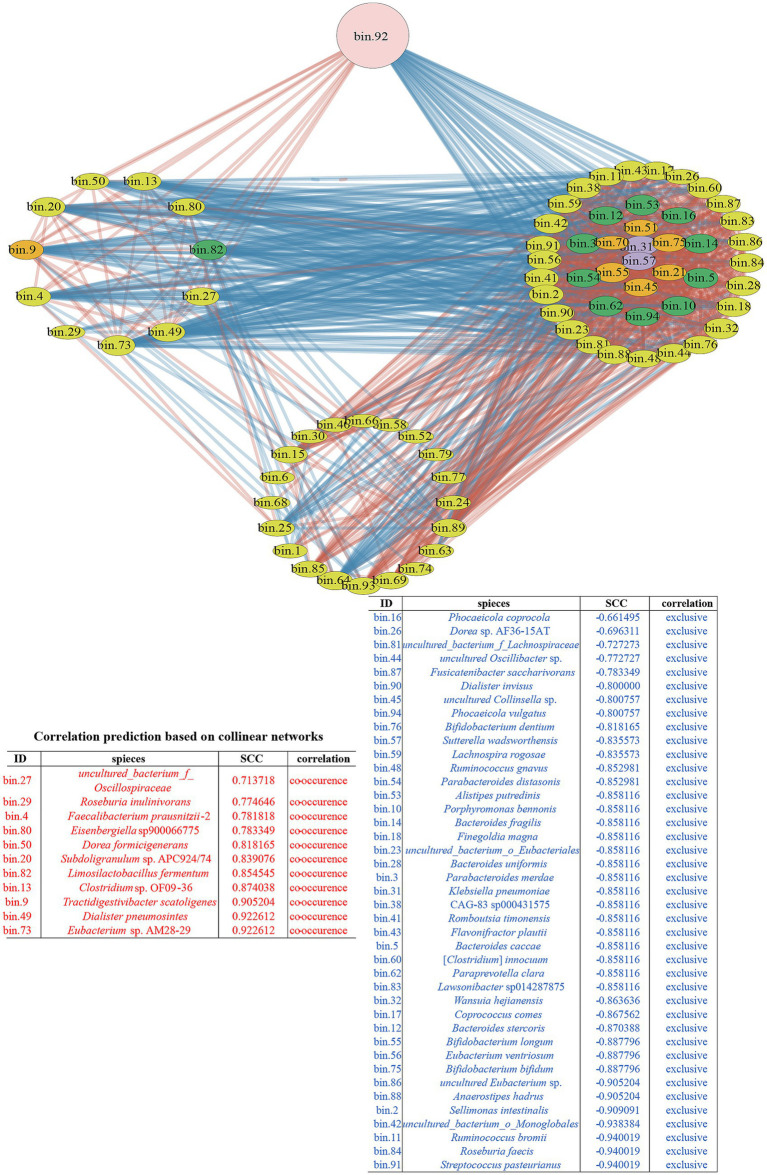
The co-occurrence network diagram with *C. minuta* SJ-2 intervention. The pink node labeled with bin.92 represented *C. minuta* SJ-2, while yellow, green, orange, and purple nodes represented Bacillota, Bacteroidetes, Actinobacteria, and Proteobacteria, respectively. The size of each node corresponded to the status of MAGs in the network, with larger nodes indicating more associations with other bacteria. The connecting lines between nodes were colored red for positive correlations and blue for negative correlations, with thicker lines reflecting stronger correlations. The right side of the network displayed bacteria indirectly associated with *C. minuta* SJ-2. The cluster at the bottom represented bacteria with no direct correlation with *C. minuta* SJ-2. The table displayed the correlation of bacteria in the co-occurrence network with *C. minuta* SJ-2: red, positive; blue, negative. All bacteria in the table were arranged in descending order of correlation intensity.

Spearman’s correlation analysis coefficient was used to construct the microbial co-occurrence network of SJ-2 with related MAGs (Oh et al., 2020). The resulting microbial network consisted of 76 nodes (MAGs) and 1,081 edges (Figure 4, Supplementary Table S12). According to the microbial network, 52 nodes were directly associated with SJ-2, among which 11 and 41 MAGs showed positive and negative correlations with SJ-2, respectively (Figure 4, Supplementary Table S13). Among the 11 MAGs exhibiting a positive correlation with SJ-2, *Eubacterium* sp. AM28-29, *D. pneumosintes* and *Tractidigestivibacter scatoligene* were predicted to have the highest correlation with SJ-2 (SCC > 0.9), *T. scatoligenes*, *Limosilactobacillus fermentum*, *F. praussnitzii* and *Roseburia inulinivorans* were of great interest as potential probiotics in previous researches (Lopez-Siles et al., 2017; Sankaranarayanan et al., 2021; Hao et al., 2023; Ozen et al., 2023). As a comparison, several species of *Bifidobacterium* and *Bacteroidetes* exhibited negative associations with SJ-2 (Figure 4, Supplementary Table S13). Additionally, we noticed several conditionally pathogenic bacteria including *Ruminococcus gnavus* (Henke et al., 2019; Zhai et al., 2023), *Bacteroides fragilis* (Goldstein et al., 2017; Zamani et al., 2019), *Finegoldia magna* (Boyanova et al., 2016), *Sutterella wadsworthensis* (Kirk et al., 2021), *Klebsiella pneumoniae* (Lee et al., 2017), and *S. pasteurianus* (Wang et al., 2022), all showed exclusionary relationships with SJ-2, which highlight potential beneficial effects of SJ-2 from host aspects.

### 3.4 Isolation and identification of gut microbes

**Figure 5:**
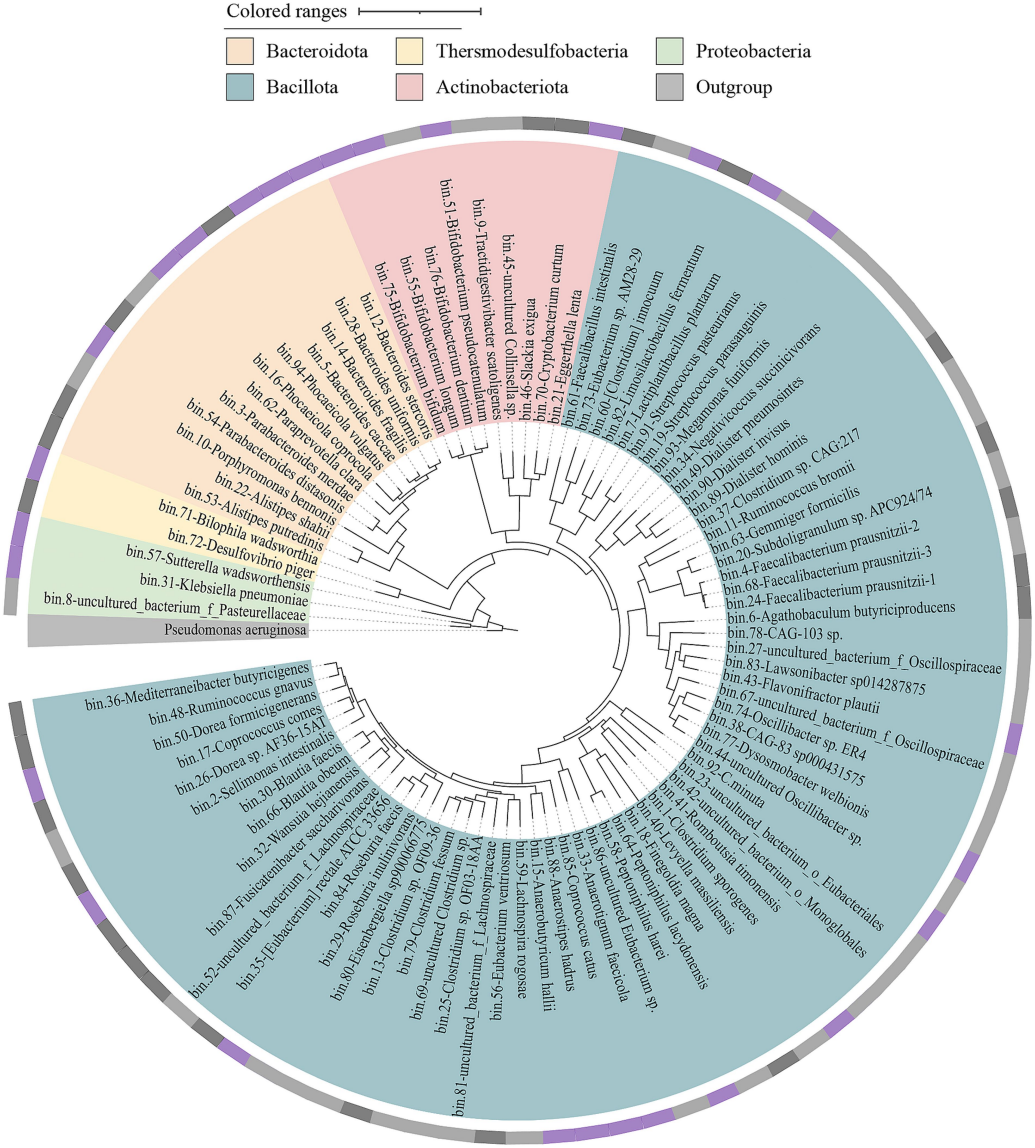
The phylogenetic tree of medium to high quality MAGs was obtained from three different groups. A phylogenetic tree was constructed based on the genome sequences of 91 MAGs using GTDBtk. The tree was built using p_Proteobacteria (GCF_000006765.1) as an outgroup to determine the evolutionary relationships among the MAGs, the outer ring represents the isolation status of the corresponding MAGs at the species level. Cycles from inside to outside: (1) phylum-level assignments (Bacteroidota, orange; Thersmodesulfobacteria, yellow; Proteobacteria, green; Actinobacteriota, pink; Bacillota, blue; Outgroup, gray); (2) isolation/culture status of the 91 (cultivated in this study, purple; strains from previous studies, black; no representative culture, gray).

**Figure 6:**
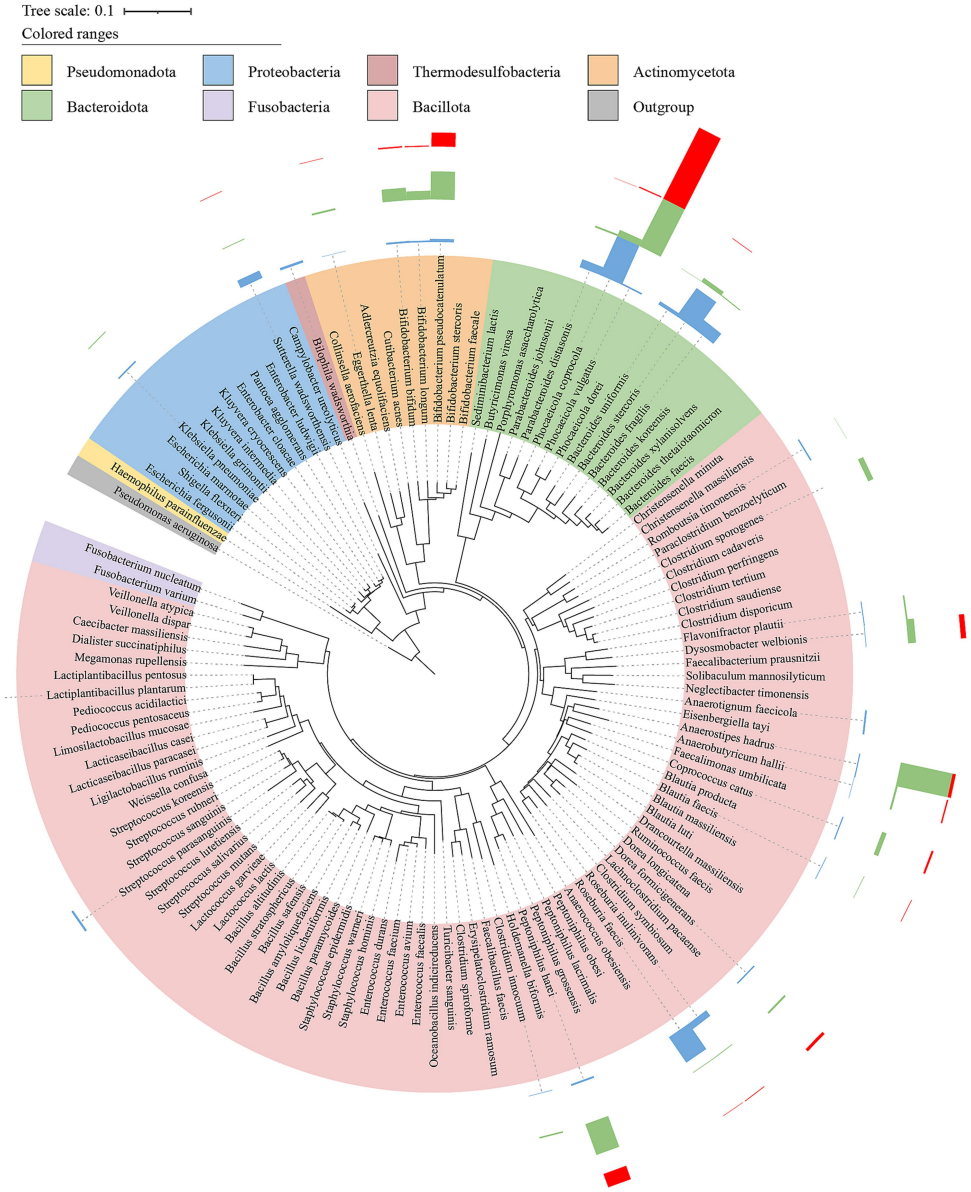
Phylogenetic tree of bacteria isolated and cultured from intestinal samples. Based on the 16S rRNA gene sequences of 121 bacterial strains, a phylogenetic tree (NJ tree) was constructed using MEGA11 with Clustal W algorithm for multiple sequence alignment, using strain *Pseudomonas aeruginosa* DSM 50071 (NR_026078.1) as an outgroup. The Bar charts were the abundance of cultivated bacterial strains corresponding to MAGs in three treatments (red, Feces; green, GAMe; blue, CMe).

Totally 1,174 bacterial colonies were isolated from fecal samples using two distinct pretreatment methods and 16 different culture conditions, and further sequenced for their 16S rRNA gene targeting regions greater than 1.4 kb to facilitate their accurate identification. The comprehensive analysis resulted in the classification of 1,174 strains into 121 distinct species, with a stringent threshold of 16S rRNA gene similarity ≥98.7%, which covered 42.6% (29 species) of all the validly named MAGs. However, there were still 22 MAGs that were not explicitly classified into validly published bacterial species. Therefore, we cannot map any isolates to them according to MAG sequences (Figure 5, Supplementary Table S14). Other than the 22 unclassified MAGs, 39 MAGs were not successfully isolated, of which some even showed high abundance in the fecal samples, such as *Megamonas funiformis*, *Roseburia faecis*, and *F. prausnitzii* (relative abundance >3%). In addition, 91 isolated bacterial species were unmapped to qualified MAGs (used for analysis), which accounted for approximately 76% of the total isolates (Figures 5, 6).

### 3.5 Validation of the predicted interactions between *Christensenella minuta* and other bacterial strains via pair-wise cocultures

**Figure 7:**
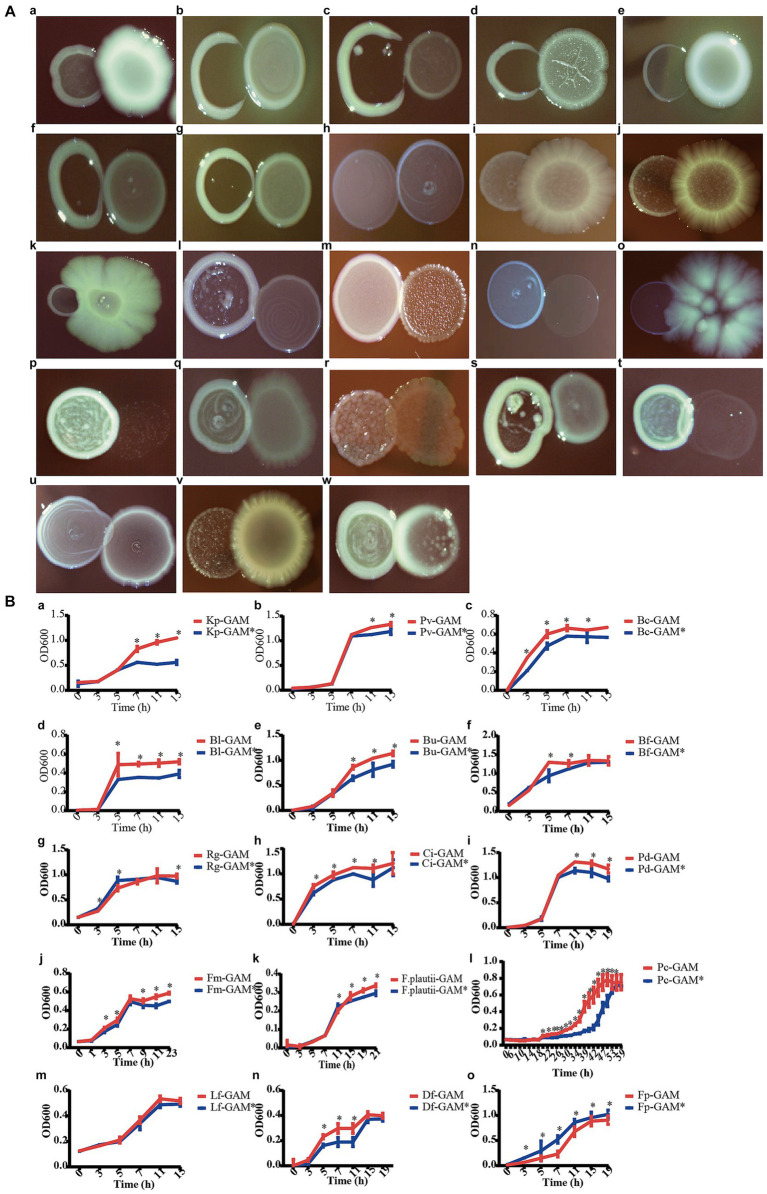
*C. minuta* SJ-2 interacts with other bacteria. **(A)** The pairwise co-cultivation on agar plates. **(B)** The growth of bacteria in mmGAM broth (red curves) and broth that was conditioned with *C. minuta* SJ-2 (blue curves). (a) *Klebsiella pneumoniae*; (b) *Phocaeicola vulgatus*; (c) *Bacteroides caccae*; (d) *Bifidobacterium bifidum*; (e) *Bifidobacterium longum*; (f) *Bacteroides uniformis*; (g) *Bacteroides fragilis*; (h) *Bacteroides stercoris*; (i) *Eubacterium ventriosum*; (j) *Ruminococcus gnavus*; (k) *Clostridium innocuum*; (l) *Parabacteroides distasonis*; (m) *Finegoldia magna*; (n) *Anaerostipes hadrus*; (o) *Romboutsia timonensis*; (p) *Alistipes putredinis*; (q) *Flavonifractor plautii*; (r) *Sellimonas intestinalis*; (s) *Parabacteroides merdae*; (t) *Phocaeicola coprocola*; (u) *Limosilactobacillus fermentum*; (v) *Dorea formicigenerans*; (w) *Faecalibacterium prausnitzii* (same number as in solid culture medium).

In order to validate the predicted correlation in the microbial network between *C. minuta* and other gut bacterial taxonomies, we tested the interaction of SJ-2 with 16 strains from the bacterial collection of this study, and 7 strains from a previous study (Liu C. et al., 2021) using two methods: (1) pair-wise coculture on agar plates, and (2) sequential cultivation of targeted strain in broth that were conditioned with SJ-2. In the pair-wise coculture, *Klebsiella pneumoniae, Phocaeicola vulgatus, Bacteroides caccae, Bifidobacterium bifidum, Bifidobacterium longum, Bacteroides uniformis, Bacteroides fragilis, Bacteroides stercoris, Eubacterium ventriosum, Ruminococcus gnavus, Clostridium innocuum* were consistently observed to compete with SJ-2, which was consistent with the prediction in the network [Figure 7A (a–k)]. However, *Parabacteroides distasonis, Finegoldia magna, Anaerostipes hadrus, Romboussia timonensis, Alistipes putredinis, Flavonifractor plautii, Sellimonas intestinalis, Paraacteroides merdae, Phocaeicola coprocola* showed neutral interactions with SJ-2, which cannot correspond their predicted negative relationships in the co-occurrence network [Figure 7A (l–t)]. Meanwhile, among the predicted positive correlated bacteria, *Limosillactobacillus fermentum* did not exhibit significant growth enhancement when co-cultured with SJ-2; *Dorea formigenes* competed with SJ-2, which were not consistent with the prediction from the co-occurrence network. Only *F. prausnitzii* showed relatively increased growth in the presence of SJ-2 [Figure 7A (u–w)]. Among all tested strains paired with SJ-2, 12 strains showing consistent tendency with the results in the co-occurrence network prediction indicated the predicted accuracy was about 52.2%.

In comparison, the results of all the liquid culture tests were almost consistent with the relationship predicted in the network except for *D. formigenes*, including *F. plautii*, *P. distasonis*, *F. magna*, and *P. coprocola*, which showed neutral relationships in plates but negative correlations in the network (Figure 7B). SJ-2 affected the bacterial growth on either biomass or growth rate or even both. For example, *B. fragilis* and *P. coprocola* had prolonged log phases under SJ-2 intervened condition compared with control [Figure 7B (g, t)] while the maximum biomass production of *K. pneumoniae*, *P. vulgatus*, *P. distasonis*, and *F. plautii* was reduced [Figure 7B (a, b, i, q)]. In contrast, both the growth rates and the maximum biomass production of *B. caccae, B. longum*, *B. uniformis*, *C. innocuum*, and *F. magna* in the conditioned medium were impaired [Figure 7B (c, e, f, k)]. On the other hand, both the growth rate and maximum biomass of *F. prausnitzii* were significantly enhanced with SJ-2 intervention confirming their positive correlation in the network [Figure 7B (w)].

### 3.6 Nutrient competition and metabolic cross-feeding contributed in negative and positive interactions, respectively, between *Christensenella minuta* and partner bacterial strains

**Figure 8:**
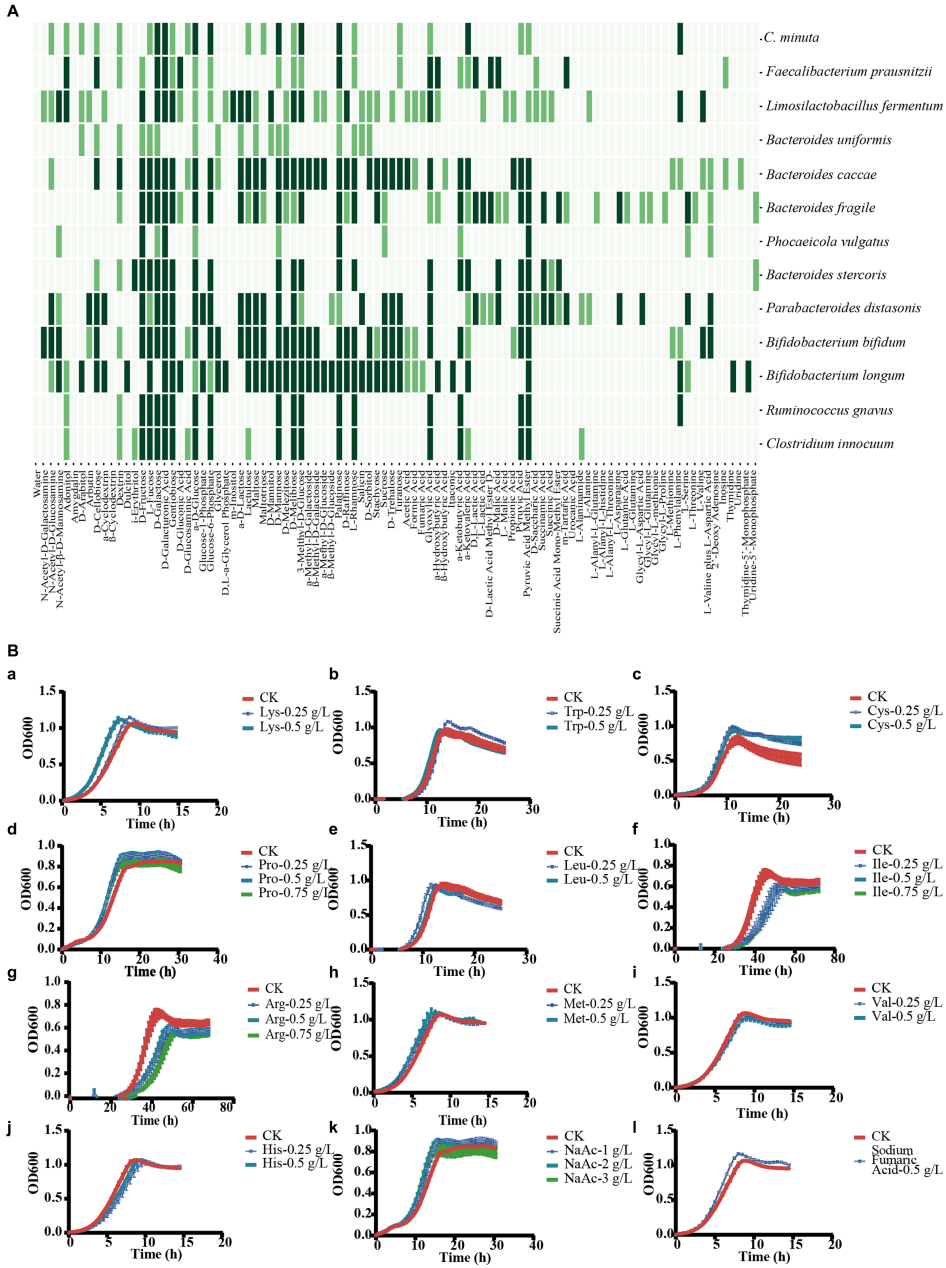
Description of the interaction mechanisms between *C. minuta* SJ-2 and other bacteria. **(A)** Assimilation of substrates. Dark green: highly utilized; light green: moderately utilized; no color: none utilized. **(B)** Substrate utilization experiment of *F. prausnitzii* to explore the mechanism of cross-feeding: (a) lysine; (b) tryptophan; (c) cysteine; (d) proline; (e) leucine; (f) isoleucine; (g) arginine; (h) methionine; (i) valine; (j) histidine; (k) sodium acetate; (l) fumaric acid.

In order to explore the interaction mechanism between *C. minuta* and the other gut bacteria, we performed genome data mining for potential metabolic crossing-feeding and conducted carbon source assimilation tests. Nutrient source assimilation tests showed that there were multiple overlaps in the majority of carbon source utilization between SJ-2 and bacteria showing negative correlation with it in the network, such as *B. caccae, B. uniformis, B. fragilis*, *B. stercoris, P. vulgatus, P. distasonis, B. longum*, *and B. bifidum* resulting in competition between them (Figure 8A). In addition, SJ-2 had a narrower carbon source utilization range than most of the competitors. For example, among all tested 95 nutrient sources, SJ-2 can utilize 25, while *B. caccae*, *B. fragilis*, *P. distasonis*, *B. bifidum*, and *B. longum* can utilize over 75% of the substances employed by SJ-2, and at least 20 more that SJ-2 cannot use.

**Figure 9:**
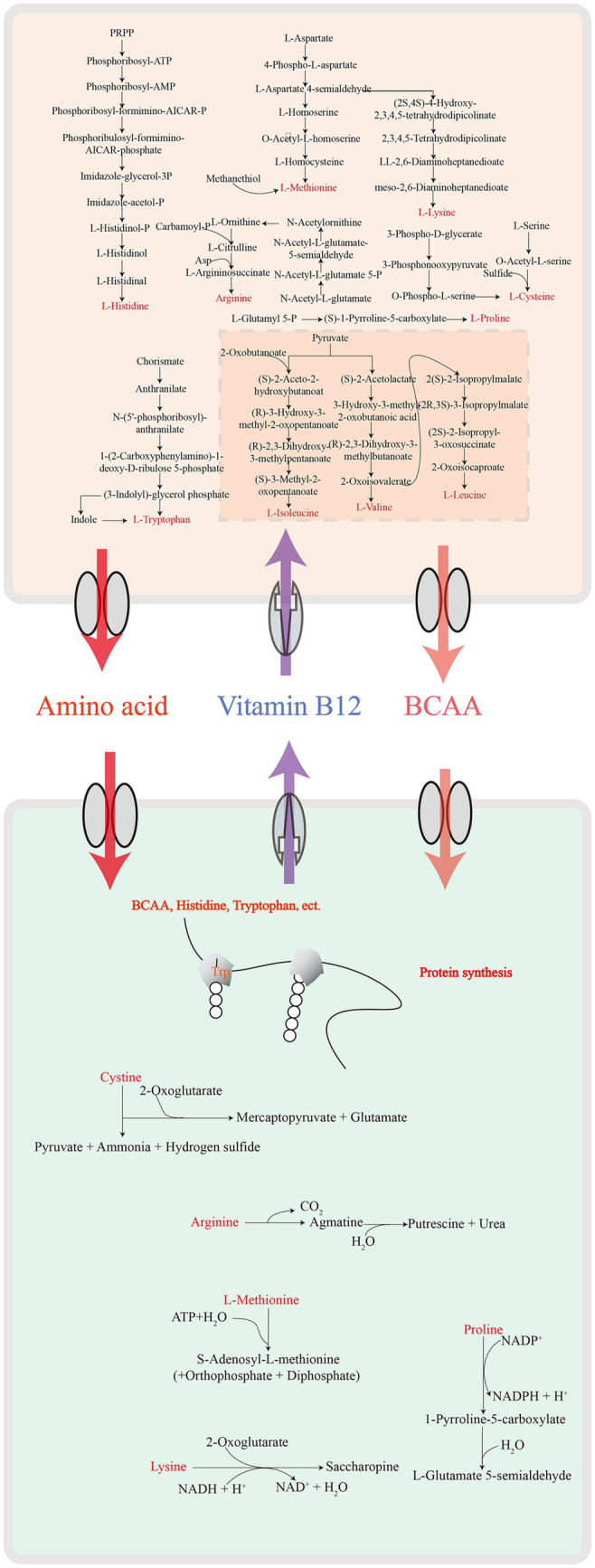
Demonstration of metabolic cross-feeding between *C. minuta* SJ-2 and *F. prausnitzii.*

To address the mechanisms of the growth enhancement of *F. prausnitzii* by SJ-2, we annotated the metabolic pathways of *F. prausnitzii* and SJ-2 for their genome sequences (Figure 9), and tested ten amino acids as well as fumaric and acetic acids that SJ-2 could apply for their effects on the growth of *F. prausnitzii* (Figure 8B). The results showed that lysine, proline, tryptophan, cysteine, sodium acetate, and fumaric acid significantly promoted the growth rate, while proline and sodium acetate even increased the biomass production of *F. prausnitzii*. We observed also that leucine, isoleucine, and arginine prolonged the generation time of *F. prausnitzii*, with the latter two potentially reducing its overall biomass (Table 1). In addition, high concentrations of methionine, valine, and histidine did not exhibit a significant effect on the growth of *F. prausnitzii*, highlighting their limited impact.

**Table 1:** Statistical table for maximum growth (absorbance at 600 nm), generation time (h), and growth rate (/h) of *F. prausnitzii* in culture medium with different doses of additives.

	Maximum biomass				Generation time				Logarithmic growth rate			
	CK	0.25 g/L	0.5 g/L	0.75 g/L	CK	0.25 g/L	0.5 g/L	0.75 g/L	CK	0.25 g/L	0.5 g/L	0.75 g/L
Lys	1.080 ± 0.019	1.132 ± 0.015	1.098 ± 0.055		1.987 ± 0.077	1.636 ± 0.070*	1.682 ± 0.107		0.353 ± 0.013	0.426 ± 0.018**	0.416 ± 0.027*	
Trp	0.952 ± 0.048	1.084 ± 0.014	0.930 ± 0.012		1.551 ± 0.016	1.423 ± 0.058*	1.616 ± 0.033		0.447 ± 0.005	0.489 ± 0.0203*	0.429 ± 0.009	
Cys	0.863 ± 0.075	0.995 ± 0.008	0.935 ± 0.026		1.882 ± 0.028	1.720 ± 0.034*	1.527 ± 0.113*		0.369 ± 0.005	0.404 ± 0.008*	0.459 ± 0.036*	
Pro	0.849 ± 0.013	0.913 ± 0.018*	0.930 ± 0.016**	0.856 ± 0.048	3.349 ± 0.087	2.836 ± 0.066*	2.927 ± 0.072*	2.958 ± 0.057*	0.208 ± 0.005	0.245 ± 0.006**	0.237 ± 0.006**	0.235 ± 0.005*
Leu	0.938 ± 0.054	0.921 ± 0.028	0.886 ± 0.025		1.486 ± 0.023	1.497 ± 0.011	1.621 ± 0.024**		0.467 ± 0.007	0.463 ± 0.003	0.428 ± 0.006*	
Ile	0.753 ± 0.027	0.616 ± 0.008*	0.615 ± 0.0110*	0.516 ± 0.030**	4.471 ± 0.309	7.247 ± 0.122**	6.640 ± 0.194**	5.721 ± 0.433*	0.160 ± 0.012	0.096 ± 0.002**	0.105 ± 0.003*	0.123 ± 0.011
Arg	0.753 ± 0.027	0.604 ± 0.014**	0.602 ± 0.027*	0.552 ± 0.014**	4.471 ± 0.309	6.686 ± 0.201**	6.054 ± 0.397*	5.074 ± 0.189	0.160 ± 0.0117	0.104 ± 0.003**	0.115 ± 0.007*	0.137 ± 0.005
Met	1.063 ± 0.017	1.077 ± 0.017	1.094 ± 0.013		1.987 ± 0.077	2.108 ± 0.081	1.737 ± 0.146		0.353 ± 0.013	0.330 ± 0.013	0.404 ± 0.031	
Val	1.063 ± 0.017	1.046 ± 0.018	1.005 ± 0.043		1.987 ± 0.077	2.095 ± 0.011	2.198 ± 0.056		0.353 ± 0.013	0.331 ± 0.002	0.316 ± 0.008	
His	1.063 ± 0.017	1.033 ± 0.038	1.070 ± 0.008		1.987 ± 0.077	2.243 ± 0.122	2.192 ± 0.008		0.363 ± 0.016	0.312 ± 0.017	0.317 ± 0.008	
NaAc	0.849 ± 0.013	0.888 ± 0.021	0.914 ± 0.004*	0.765 ± 0.073	3.349 ± 0.087	2.954 ± 0.112*	2.943 ± 0.020*	3.202 ± 0.230	0.208 ± 0.005	0.236 ± 0.010*	0.236 ± 0.002*	0.219 ± 0.014
Fumarate	1.814 ± 0.743	1.166 ± 0.007			1.987 ± 0.0767	1.559 ± 0.007**			0.353 ± 0.077	0.445 ± 0.007**		

Significant difference *p < 0.05, **p < 0.01.

## 4 Discussion

The intestinal microbial community is a complex of beneficial, neutral and potentially pathogenic microorganisms. The intestinal microbial homeostasis was attributed from the interaction of all gut microbes as a network. *Christensenella minuta* as a potential probiotic increasingly gained attention for its beneficial effects in weight control as well as blood sugar remaining (Kropp et al., 2021; Mazier et al., 2021; Akbuğa-Schön et al., 2023). Based on the current research findings, the colonization of *C. minuta* has a significant impact on *Oscillospira* spp., Prevotellaceae, Lactobacillaceae, Erysipelotrichaceae, and Bifidobacteriaceae, which helps in correcting the intestinal microbial displacement caused by obesity (Goodrich et al., 2014; Mazier et al., 2021). However, its interaction with other gut bacteria was rarely explored. In this study, we investigated the interaction of a *C. minuta* strain SJ-2 with multiple gut bacteria via analyzing metagenomic data collected from *in vitro* enrichment culture of fecal microbiota, and validated the prediction through one-to-one culturing experiments. After enriched treatment, fecal samples gained noticeable changes in both microbial composition and structure, especially a decrease in biodiversity. The within-sample diversity reflected by alpha diversity was decreased attributing to multiple factors. In addition to the prior microbial disturbance caused by the medium (mmGAM) enrichment, the intervention with SJ-2 further reduced the microbial diversity, particularly for Bacteroidaceae, Selenomonadaceae, and Rikenellaceae. It was not surprising that the presence of SJ-2 consumed some nutrients partially responsible for a discernible decrease in microbial diversity, as evidenced by the high overlap observed between SJ-2 and competitive bacteria such as Bacteroides in the carbon metabolism and substrate utilization investigation. However, some bacterial groups specifically changed with the intervention of SJ-2, such as Lactobacillaceae, Oscillospiraceae, Ruminococcaceae, Atopobiaceae (increased), and Streptococcaceae (decreased), which revealed the specific effects of SJ-2 on fecal microbial community not only realized on the nutrient deficiency. The co-occurrence network analysis results also confirmed that SJ-2 occupied a critical central position that highly associated with most of the gut bacteria, which was consistent with previous studies (Goodrich et al., 2014; Mazier et al., 2021).

Forty MAGs were specifically reduced by SJ-2 in the co-occurrence network, including several opportunistic pathogens such as *Finegoldia magna* (Shetty et al., 2023), *Klebsiella pneumoniae* (Lee et al., 2017), *Bacteroides fragilis* (Goldstein et al., 2017), *Bacteroides caccae* (Wei et al., 2001; Guo et al., 2016), and *Clostridium innocuum* (Chia et al., 2017). This was confirmed by functional pathway analysis: the pathogenicity-related genes were reduced in CMe samples. Among all the bacteria that were confirmed to compete with SJ-2, only *C. innocuum* showed vulnerability in the competition. *C. innocuum* survives better in lipid-rich environments, and results in creeping fat which is a mysterious feature of Cron’s disease (Ha et al., 2020). These combined results reveal that *C. minuta*’s ability for lipid metabolism may contribute to inhibiting the growth of *C. innocuum*. Furthermore, this result highlights inhibiting the colonization and function of obesity and intestinal disease promoting microorganisms may be one contributor to *C. minuta*’s promotion of weight loss and prevention of intestinal diseases.

In addition, the abundance of the Streptococcaceae family exhibited a specific decrease, with *Streptococcus pasteurianus* showing the strongest negative correlation with SJ-2 in the co-occurrence network. *S. pasteurianus* is a potential pathogen for inflammatory diseases, which is known to be enriched in Crohn’s disease but reduced as SCFAs accumulated (Nagayama et al., 2020; Wang et al., 2020; Ma et al., 2023). Therefore, the negative correlation between SJ-2 and *S. pasteurianus* may be attributed to the efficient SCFAs production of *C. minuta* (Kropp et al., 2021). This finding offers a new perspective on the development of *C. minuta* as a probiotic agent for the regulation of inflammatory bowel diseases. Carbon utilization is overlapped between SJ-2 and various species in the genera *Bacteroides* and *Bifidobacterium*, such as *Bacteroides uniformis, Bacteroides stercoris, Bifidobacterium longum*, and *Bifidobacterium bifidum*, indicating their negative correlation in the network. *Bacteroides* spp. and *Bifidobacterium* spp. are the major commensal bacteria in human gut, most of which show beneficial effects for human health (Zafar and Saier, 2021; Derrien et al., 2022). Although it cannot be concluded that their negative correlation with *C. minuta* may be harmful to human health, the competition for carbon sources between *C. minuta* and *Bacteroides* spp. or *Bifidobacterium* spp. may result in *C. minuta*’s abundance at a low level in human gut. In fact, previous studies show that *B. uniformis* is negatively correlated with *C. minuta* (Upadhyaya et al., 2016), which is highly heritable, although accounts for a low proportion of the fecal microbiota (Waters and Ley, 2019; Mazier et al., 2021). Stable colonization has been a challenge for applying most probiotic products. According to the results of this study, a combination of probiotic bacteria such as *C. minuta* with one or several bacterial strains showing cross-feeding ability may be an option to enhance the colonization and subsequently improve probiotic functions in the human bodies.

*Faecalibacterium prausnitzii*, which is among the 11 enriched species in the CMe samples, is a potential next-generation probiotic for high butyrate production, anti-inflammation, and prevention of intestinal pathogens (Sokol et al., 2008; Bai et al., 2022). In this study, we demonstrated that SJ-2 cross-feeds *F. prausnitzii* via producing exogenous acetate, cysteine, proline, and lysine, which were required for the fermentation and reproduction of *F. prausnitzii*. This phenomenon is consistent with Otten *et al*’s results (Otten et al., 2021), which support the mechanism of the dependency effect of *F. prausnitzii* on exogenous acetic acid (Duncan et al., 2004).

The intervention of SJ-2 increased the abundance of many potential beneficial bacteria in the family Lactobacillaceae, Oscillospiraceae, Ruminococcaceae, and Atopobiaceae, such as *Limosilactobacillus fermentum, F. prausnitzii*, and *Tractidigestivibacter scatoligenes* which strongly revealed their role in host carbohydrate and lipid metabolism regulations. *Lactobacillus* spp. have been widely used to improve metabolic diseases, enhance gut immunity, and protect gut barrier integrity (Huang et al., 2022). Moreover, members in the Oscillospiraceae and Ruminococcaceae families produce high levels of SCFAs through carbohydrate fermentation, participating in mediating immune response and inducing regulatory T-cell formation (Rooks and Garrett, 2016). In addition, their negative correlation with body weight and inflammatory markers indicated their positive role in host metabolism regulation and anti-inflammatory function (Kim et al., 2020; Hu et al., 2021; Leth et al., 2023). Members of Atopobiaceae repress the colonization of drug-resistant bacteria in elderly individuals (Ducarmon et al., 2021; Morinaga et al., 2022). Fascinatingly, most of these bacteria contain bile salt hydrolase (BSH) enzymes (EC3.5.1.24) which are involved in antibacterial activity, lipid absorption regulation, and cholesterol reduction processes, akin to *C. minuta* (Lee et al., 2020; Morinaga et al., 2022; Yang and Wu, 2022; Lin et al., 2023). The positive association between *C. minuta* and those beneficial bacteria demonstrated that in addition to direct involvement in host metabolism, *C. minuta* can regulate host’s metabolic dysfunctions through collaboration with other beneficial commensal bacteria (Miquel et al., 2013; Leylabadlo et al., 2020; Wu et al., 2021).

Despite significant changes in the microbial diversity, the fundamental metabolic functions remained stable within the community, related to amino acids, carbohydrates, cofactors and vitamins metabolisms. The intervention of SJ-2 modified the mode of metabolisms of the fecal microbial community, enhancing the transport and synthesis of glutamate, branched-chain amino acid, and polar amino acids, including tyrosine and vitamins B12, and B1. Conversely, it reduced the synthesis pathways of glutathione, methionine, tryptophan, and branched-chain amino acids. Genomic sequence analysis led to the hypothesis that SJ-2 could promote nutritional synergy by cross-feeding with other enriched bacteria. Notably, among SJ-2-enhanced metabolites, biotin is negatively correlated with BMI and is involved in regulating carbohydrate and lipid metabolism, fatty acid synthesis, and amino acid decomposition (Peterson et al., 2020), while thiamin and ascorbic acid are involved in maintaining host immune metabolism, inhibiting oxidative stress-induced NF-κB activation and pro-inflammatory cytokine release, preserving epithelial cell integrity, and reducing host susceptibility (Yadav et al., 2010; Di Renzo et al., 2020). The enrichment of coenzyme A suggests an active β-oxidation process, leading to more ketone bodies and the conversion of acetyl coenzyme A (Canibe et al., 2003). These findings suggest a high level of lipolytic activity in the community, thereby indirectly reflecting the intervention capability (Basolo et al., 2022) and the development potential of *C. minuta* in lipid metabolism. Moreover, the decreased pathogenicity and dropped abundance of opportunistic pathogens, along with the regulation of other beneficial bacteria, are closely linked to the improvement in the overall health direction of the community. As a result, the microbiota may trend toward a more favorable state for human health.

In summary, we demonstrated the interaction and dynamics between *C. minuta* SJ-2 and various gut bacteria and tried to explore the metabolic interplay particularly in the cross-feeding between *C. minuta* SJ-2 and *F. prausnitzii*. SJ-2 facilitated *F. prausnitzii*’s growth by supplying essential amino acids (lysine, proline, tryptophan, cysteine), acetic acid, and fumaric acid, while reciprocally benefiting from the latter’s biosynthesis of nutrients critical for its proliferation (Figure 9). These results enhanced our understanding of *C. minuta*’s probiotic functions and informed strategies for its application. However, this study’s limitation to a single sample underscores the necessity for broader investigation involving diverse populations to generalize these interactions. Future work should encompass sampling multiple times throughout the cultivation period to map temporal changes in microbial interactions and explore the influence of varied culture media on these dynamics. In-depth studies at the genetic level will further our understanding of the specific molecular mechanisms underpinning the symbiotic relationships observed.

## Data availability statement

The data presented in the study are deposited in the National Microbiology Data Center (NMDC: https://nmdc.cn/) with the accession number NMDC10018531.

## Ethics statement

The studies involving humans were approved by Research Ethics Committee of the Institute of Microbiology, Chinese Academy of Science. The studies were conducted in accordance with the local legislation and institutional requirements. The participants provided their written informed consent to participate in this study.

## Author contributions

CX: Conceptualization, Data curation, Formal analysis, Investigation, Methodology, Supervision, Validation, Visualization, Writing – original draft. HJ: Conceptualization, Funding acquisition, Methodology, Project administration, Supervision, Visualization, Writing – original draft, Writing – review & editing. L-JF: Data curation, Investigation, Resources, Writing – original draft. M-ZJ: Formal analysis, Methodology, Resources, Software, Writing – original draft. Y-LW: Formal analysis, Methodology, Software, Visualization, Writing – original draft. S-JL: Conceptualization, Funding acquisition, Methodology, Project administration, Resources, Supervision, Writing – review & editing.

## Footnotes

^1^https://github.com/wwood/CoverM

^2^https://github.com/geronimp/enrichM

^4^https://www.ezbiocloud.net/

^5^https://www.ncbi.nlm.nih.gov/

## References

[ref1] Abdugheni R. Wang W.-Z. Wang Y.-J. Du M.-X. Liu F.-L. Zhou N.. (2022). Metabolite profiling of human-originated Lachnospiraceae at the strain level. iMETA 1:e58. doi: 10.1002/imt2.58 PMC10989990 38867908

[ref2] Akbuğa-Schön T. Suzuki T. A. Jakob D. Vu D. L. Waters J. L. Ley R. E. (2023). The keystone gut species *Christensenella minuta* boosts gut microbial biomass and voluntary physical activity in mice. MBio:e0283623. doi: 10.1128/mbio.02836-23 [Epub ahead of print]., PMID: 38132571 PMC10865807

[ref3] Alneberg J. Bjarnason B. S. de Bruijn I. Schirmer M. Quick J. Ijaz U. Z.. (2014). Binning metagenomic contigs by coverage and composition. Nat. Methods 11, 1144–1146. doi: 10.1038/nmeth.3103, PMID: 25218180

[ref4] Bäckhed F. Roswall J. Peng Y. Feng Q. Jia H. Kovatcheva-Datchary P.. (2015). Dynamics and stabilization of the human gut microbiome during the first year of life. Cell Host Microbe 17, 690–703. doi: 10.1016/j.chom.2015.04.004 25974306

[ref5] Bai Z. Zhang N. Jin Y. Chen L. Mao Y. Sun L.. (2022). Comprehensive analysis of 84 *Faecalibacterium prausnitzii* strains uncovers their genetic diversity, functional characteristics, and potential risks. Front. Cell. Infect. Microbiol. 12:919701. doi: 10.3389/fcimb.2022.919701, PMID: 36683686 PMC9846645

[ref6] Basolo A. Magno S. Santini F. Ceccarini G. (2022). Ketogenic diet and weight loss: is there an effect on energy expenditure? Nutrients 14:1814. doi: 10.3390/nu14091814 35565778 PMC9105638

[ref7] Botté E. S. Bennett H. Engelberts J. P. Thomas T. Bell J. J. Webster N. S.. (2023). Future Ocean conditions induce necrosis, microbial dysbiosis and nutrient cycling imbalance in the reef sponge *Stylissa flabelliformis*. ISME Commun. 3:53. doi: 10.1038/s43705-023-00247-3, PMID: 37311801 PMC10264452

[ref8] Bowers R. M. Kyrpides N. C. Stepanauskas R. Harmon-Smith M. Doud D. Reddy T. B. K.. (2017). Minimum information about a single amplified genome (MISAG) and a metagenome-assembled genome (MIMAG) of bacteria and archaea. Nat. Biotechnol. 35, 725–731. doi: 10.1038/nbt.3893, PMID: 28787424 PMC6436528

[ref9] Boyanova L. Markovska R. Mitov I. (2016). Virulence arsenal of the most pathogenic species among the gram-positive anaerobic cocci, *Finegoldia magna*. Anaerobe 42, 145–151. doi: 10.1016/j.anaerobe.2016.10.007, PMID: 27756620

[ref10] Canibe N. Juntunen K. S. Knudsen K. E. B. Serena A. (2003). New insight into butyrate metabolism. Proc. Nutr. Soc. 62, 81–86. doi: 10.1079/PNS2002212 12740062

[ref11] Chang Y. Hou F. Pan Z. Huang Z. Han N. Bin L.. (2019). Optimization of Culturomics strategy in human fecal samples. Front. Microbiol. 10:2891. doi: 10.3389/fmicb.2019.02891, PMID: 31921067 PMC6927924

[ref12] Chaumeil P. A. Mussig A. J. Hugenholtz P. Parks D. H. (2022). GTDB-Tk v2: memory friendly classification with the genome taxonomy database. Bioinformatics 38, 5315–5316. doi: 10.1093/bioinformatics/btac672, PMID: 36218463 PMC9710552

[ref13] Chen Y. Chen Y. Shi C. Huang Z. Zhang Y. Li S.. (2018). SOAPnuke: a MapReduce acceleration-supported software for integrated quality control and preprocessing of high-throughput sequencing data. Gigascience 7, 1–6. doi: 10.1093/gigascience/gix120 PMC5788068 29220494

[ref14] Chen T. Liu Y.-X. Huang L. (2022). ImageGP: An easy-to-use data visualization web server for scientific researchers. iMeta 1:e5. doi: 10.1002/imt2.5 PMC10989750 38867732

[ref15] Chia J. H. Feng Y. Su L. H. Wu T. L. Chen C. L. Liang Y. H.. (2017). *Clostridium innocuum* is a significant vancomycin-resistant pathogen for extraintestinal clostridial infection. Clin. Microbiol. Infect. 23, 560–566. doi: 10.1016/j.cmi.2017.02.025, PMID: 28254687

[ref16] Chu N. D. Crothers J. W. Nguyen L. T. T. Kearney S. M. Smith M. B. Kassam Z.. (2021). Dynamic colonization of microbes and their functions after fecal microbiota transplantation for inflammatory bowel disease. MBio 12:e0097521. doi: 10.1128/mBio.00975-21 34281401 PMC8406238

[ref17] de Vos W. M. Tilg H. Van Hul M. Cani P. D. (2022). Gut microbiome and health: mechanistic insights. Gut 71, 1020–1032. doi: 10.1136/gutjnl-2021-326789, PMID: 35105664 PMC8995832

[ref18] de Vos M. G. J. Zagorski M. McNally A. Bollenbach T. (2017). Interaction networks, ecological stability, and collective antibiotic tolerance in polymicrobial infections. Proc. Natl. Acad. Sci. USA 114, 10666–10671. doi: 10.1073/pnas.1713372114, PMID: 28923953 PMC5635929

[ref19] Derrien M. Turroni F. Ventura M. van Sinderen D. (2022). Insights into endogenous Bifidobacterium species in the human gut microbiota during adulthood. Trends Microbiol. 30, 940–947. doi: 10.1016/j.tim.2022.04.004, PMID: 35577716

[ref20] Di Renzo L. Gualtieri P. Pivari F. Soldati L. Attinà A. Leggeri C.. (2020). COVID-19: is there a role for immunonutrition in obese patient? J. Transl. Med. 18:415. doi: 10.1186/s12967-020-02594-4, PMID: 33160363 PMC7647877

[ref21] Dominguez-Bello M. G. Godoy-Vitorino F. Knight R. Blaser M. J. (2019). Role of the microbiome in human development. Gut 68, 1108–1114. doi: 10.1136/gutjnl-2018-317503 30670574 PMC6580755

[ref22] Ducarmon Q. R. Terveer E. M. Nooij S. Bloem M. N. Vendrik K. E. W. Caljouw M. A. A.. (2021). Microbiota-associated risk factors for asymptomatic gut colonisation with multi-drug-resistant organisms in a Dutch nursing home. Genome Med. 13:54. doi: 10.1186/s13073-021-00869-z, PMID: 33827686 PMC8028076

[ref23] Duncan S. H. Holtrop G. Lobley G. E. Calder A. G. Stewart C. S. Flint H. J. (2004). Contribution of acetate to butyrate formation by human faecal bacteria. Br. J. Nutr. 91, 915–923. doi: 10.1079/bjn20041150 15182395

[ref24] Fan L. Peng W. Duan H. Lü F. Zhang H. He P. (2023). Presence and role of viruses in anaerobic digestion of food waste under environmental variability. Microbiome 11:170. doi: 10.1186/s40168-023-01585-z, PMID: 37537690 PMC10401857

[ref25] Faust K. Raes J. (2012). Microbial interactions: from networks to models. Nat. Rev. Microbiol. 10, 538–550. doi: 10.1038/nrmicro2832 22796884

[ref26] Federhen S. (2012). The NCBI taxonomy database. Nucleic Acids Res. 40, D136–D143. doi: 10.1093/nar/gkr1178, PMID: 22139910 PMC3245000

[ref27] Fu J. Bonder M. J. Cenit M. C. Tigchelaar E. F. Maatman A. Dekens J. A.. (2015). The gut microbiome contributes to a substantial proportion of the variation in blood lipids. Circ. Res. 117, 817–824. doi: 10.1161/circresaha.115.306807, PMID: 26358192 PMC4596485

[ref28] Goldstein E. J. Citron D. M. Tyrrell K. L. Leoncio E. S. Merriam C. V. (2017). The underappreciated *in vitro* activity of tedizolid against *Bacteroides fragilis* species, including strains resistant to metronidazole and carbapenems. Anaerobe 43, 1–3. doi: 10.1016/j.anaerobe.2016.09.008, PMID: 27713022

[ref29] Goodrich J. K. Waters J. L. Poole A. C. Sutter J. L. Koren O. Blekhman R.. (2014). Human genetics shape the gut microbiome. Cell 159, 789–799. doi: 10.1016/j.cell.2014.09.053, PMID: 25417156 PMC4255478

[ref30] Guo Z. Zhang J. Wang Z. Ang K. Y. Huang S. Hou Q.. (2016). Intestinal microbiota distinguish gout patients from healthy humans. Sci. Rep. 6:20602. doi: 10.1038/srep20602, PMID: 26852926 PMC4757479

[ref31] Ha C. W. Y. Martin A. Sepich-Poore G. D. Shi B. Wang Y. Gouin K.. (2020). Translocation of viable gut microbiota to mesenteric adipose drives formation of creeping fat in humans. Cell 183, 666–683.e17. doi: 10.1016/j.cell.2020.09.009, PMID: 32991841 PMC7521382

[ref32] Hao Z. Meng C. Li L. Feng S. Zhu Y. Yang J.. (2023). Positive mood-related gut microbiota in a long-term closed environment: a multiomics study based on the "lunar palace 365" experiment. Microbiome 11:88. doi: 10.1186/s40168-023-01506-0, PMID: 37095530 PMC10124008

[ref33] Henke M. T. Kenny D. J. Cassilly C. D. Vlamakis H. Xavier R. J. Clardy J. (2019). *Ruminococcus gnavus*, a member of the human gut microbiome associated with Crohn's disease, produces an inflammatory polysaccharide. Proc. Natl. Acad. Sci. USA 116, 12672–12677. doi: 10.1073/pnas.1904099116, PMID: 31182571 PMC6601261

[ref34] Hibbing M. E. Fuqua C. Parsek M. R. Peterson S. B. (2010). Bacterial competition: surviving and thriving in the microbial jungle. Nat. Rev. Microbiol. 8, 15–25. doi: 10.1038/nrmicro2259, PMID: 19946288 PMC2879262

[ref35] Hiseni P. Rudi K. Wilson R. C. Hegge F. T. Snipen L. (2021). HumGut: a comprehensive human gut prokaryotic genomes collection filtered by metagenome data. Microbiome 9:165. doi: 10.1186/s40168-021-01114-w, PMID: 34330336 PMC8325300

[ref36] Hu Q. Niu Y. Yang Y. Mao Q. Lu Y. Ran H.. (2021). Polydextrose alleviates adipose tissue inflammation and modulates the gut microbiota in high-fat diet-fed mice. Front. Pharmacol. 12:795483. doi: 10.3389/fphar.2021.795483, PMID: 35185543 PMC8848743

[ref37] Huang R. Wu F. Zhou Q. Wei W. Yue J. Xiao B.. (2022). *Lactobacillus* and intestinal diseases: mechanisms of action and clinical applications. Microbiol. Res. 260:127019. doi: 10.1016/j.micres.2022.127019, PMID: 35421680

[ref38] Jiang M. Z. Zhu H. Z. Zhou N. Liu C. Jiang C. Y. Wang Y.. (2022). Droplet microfluidics-based high-throughput bacterial cultivation for validation of taxon pairs in microbial co-occurrence networks. Sci. Rep. 12:18145. doi: 10.1038/s41598-022-23000-7, PMID: 36307549 PMC9616874

[ref39] Kanehisa M. Goto S. (2000). KEGG: Kyoto encyclopedia of genes and genomes. Nucleic Acids Res. 28, 27–30. doi: 10.1093/nar/28.1.27, PMID: 10592173 PMC102409

[ref40] Kang D. D. Li F. Kirton E. Thomas A. Egan R. An H.. (2019). MetaBAT 2: an adaptive binning algorithm for robust and efficient genome reconstruction from metagenome assemblies. PeerJ 7:e7359. doi: 10.7717/peerj.7359, PMID: 31388474 PMC6662567

[ref41] Kim M. H. Yun K. E. Kim J. Park E. Chang Y. Ryu S.. (2020). Gut microbiota and metabolic health among overweight and obese individuals. Sci. Rep. 10:19417. doi: 10.1038/s41598-020-76474-8, PMID: 33173145 PMC7655835

[ref42] Kirk K. F. Andersen K. L. Tarpgaard I. H. Nielsen H. L. (2021). Three cases of *Sutterella wadsworthensis* bacteremia secondary to abdominal infections. Anaerobe 72:102460. doi: 10.1016/j.anaerobe.2021.102460, PMID: 34563694

[ref43] Klimenko N. S. Tyakht A. V. Popenko A. S. Vasiliev A. S. Altukhov I. A. Ischenko D. S.. (2018). Microbiome responses to an uncontrolled short-term diet intervention in the frame of the citizen science project. Nutrients 10:576. doi: 10.3390/nu10050576, PMID: 29738477 PMC5986456

[ref44] Kropp C. Le Corf K. Relizani K. Tambosco K. Martinez C. Chain F.. (2021). The keystone commensal bacterium *Christensenella minuta* DSM 22607 displays anti-inflammatory properties both *in vitro* and *in vivo*. Sci. Rep. 11:11494. doi: 10.1038/s41598-021-90885-1, PMID: 34075098 PMC8169850

[ref45] Lagier J. C. Dubourg G. Million M. Cadoret F. Bilen M. Fenollar F.. (2018). Culturing the human microbiota and culturomics. Nat. Rev. Microbiol. 16, 540–550. doi: 10.1038/s41579-018-0041-0 29937540

[ref46] Langmead B. Trapnell C. Pop M. Salzberg S. L. (2009). Ultrafast and memory-efficient alignment of short DNA sequences to the human genome. Genome Biol. 10:R25. doi: 10.1186/gb-2009-10-3-r25, PMID: 19261174 PMC2690996

[ref47] Lee C. R. Lee J. H. Park K. S. Jeon J. H. Kim Y. B. Cha C. J.. (2017). Antimicrobial resistance of Hypervirulent *Klebsiella pneumoniae*: epidemiology, Hypervirulence-associated determinants, and resistance mechanisms. Front. Cell. Infect. Microbiol. 7:483. doi: 10.3389/fcimb.2017.00483, PMID: 29209595 PMC5702448

[ref48] Lee G. You H. J. Bajaj J. S. Joo S. K. Yu J. Park S.. (2020). Distinct signatures of gut microbiome and metabolites associated with significant fibrosis in non-obese NAFLD. Nat. Commun. 11:4982. doi: 10.1038/s41467-020-18754-5, PMID: 33020474 PMC7536225

[ref49] Lee-Sarwar K. A. Kelly R. S. Lasky-Su J. Zeiger R. S. O'Connor G. T. Sandel M. T.. (2019). Integrative analysis of the intestinal metabolome of childhood asthma. J. Allergy Clin. Immunol. 144, 442–454. doi: 10.1016/j.jaci.2019.02.032, PMID: 30914378 PMC6688902

[ref50] Leth M. L. Pichler M. J. Abou Hachem M. (2023). Butyrate-producing colonic clostridia: picky glycan utilization specialists. Essays Biochem. 67, 415–428. doi: 10.1042/ebc20220125, PMID: 36350044

[ref51] Letunic I. Bork P. (2021). Interactive tree of life (iTOL) v5: an online tool for phylogenetic tree display and annotation. Nucleic Acids Res. 49, W293–w296. doi: 10.1093/nar/gkab301, PMID: 33885785 PMC8265157

[ref52] Leylabadlo H. E. Ghotaslou R. Feizabadi M. M. Farajnia S. Moaddab S. Y. Ganbarov K.. (2020). The critical role of *Faecalibacterium prausnitzii* in human health: An overview. Microb. Pathog. 149:104344. doi: 10.1016/j.micpath.2020.104344, PMID: 32534182

[ref53] Li W. Godzik A. (2006). Cd-hit: a fast program for clustering and comparing large sets of protein or nucleotide sequences. Bioinformatics 22, 1658–1659. doi: 10.1093/bioinformatics/btl158 16731699

[ref54] Li X. Li Z. He Y. Li P. Zhou H. Zeng N. (2020). Regional distribution of Christensenellaceae and its associations with metabolic syndrome based on a population-level analysis. PeerJ 8:e9591. doi: 10.7717/peerj.9591, PMID: 32832265 PMC7413085

[ref55] Lin L. Lai Z. Yang H. Zhang J. Qi W. Xie F.. (2023). Genome-centric investigation of bile acid metabolizing microbiota of dairy cows and associated diet-induced functional implications. ISME J. 17, 172–184. doi: 10.1038/s41396-022-01333-5, PMID: 36261508 PMC9750977

[ref56] Liu C. Du M. X. Abuduaini R. Yu H. Y. Li D. H. Wang Y. J.. (2021). Enlightening the taxonomy darkness of human gut microbiomes with a cultured biobank. Microbiome 9:119. doi: 10.1186/s40168-021-01064-3, PMID: 34020714 PMC8140505

[ref57] Liu P. Zhang T. Zheng Y. Li Q. Su T. Qi Q. (2021). Potential one-step strategy for PET degradation and PHB biosynthesis through co-cultivation of two engineered microorganisms. Eng. Microbiol. 1:100003. doi: 10.1016/j.engmic.2021.100003 PMC11610943 39629164

[ref58] Lopez-Siles M. Duncan S. H. Garcia-Gil L. J. Martinez-Medina M. (2017). *Faecalibacterium prausnitzii*: from microbiology to diagnostics and prognostics. ISME J. 11, 841–852. doi: 10.1038/ismej.2016.176, PMID: 28045459 PMC5364359

[ref59] Ma M. Wang S. Zhu X. Li X. Bao Y. Chen X.. (2023). The identification of *Streptococcus pasteurianus* obtained from six regions in China by multiplex PCR assay and the characteristics of pathogenicity and antimicrobial resistance of this zoonotic pathogen. Pathogens 12:615. doi: 10.3390/pathogens12040615, PMID: 37111501 PMC10142533

[ref60] Ma C. Wasti S. Huang S. Zhang Z. Mishra R. Jiang S.. (2020). The gut microbiome stability is altered by probiotic ingestion and improved by the continuous supplementation of galactooligosaccharide. Gut Microbes 12:1785252. doi: 10.1080/19490976.2020.1785252, PMID: 32663059 PMC7524268

[ref61] Mac Aogáin M. Narayana J. K. Tiew P. Y. Ali N. Yong V. F. L. Jaggi T. K.. (2021). Integrative microbiomics in bronchiectasis exacerbations. Nat. Med. 27, 688–699. doi: 10.1038/s41591-021-01289-7, PMID: 33820995

[ref62] Mazier W. Le Corf K. Martinez C. Tudela H. Kissi D. Kropp C.. (2021). A new strain of *Christensenella minuta* as a potential biotherapy for obesity and associated metabolic diseases. Cell 10:823. doi: 10.3390/cells10040823, PMID: 33917566 PMC8067450

[ref63] Miquel S. Martín R. Rossi O. Bermúdez-Humarán L. G. Chatel J. M. Sokol H.. (2013). *Faecalibacterium prausnitzii* and human intestinal health. Curr. Opin. Microbiol. 16, 255–261. doi: 10.1016/j.mib.2013.06.003 23831042

[ref64] Morinaga K. Kusada H. Tamaki H. (2022). Bile salt hydrolases with extended substrate specificity confer a high level of resistance to bile toxicity on Atopobiaceae Bacteria. Int. J. Mol. Sci. 23:10980. doi: 10.3390/ijms231810980, PMID: 36142891 PMC9506489

[ref65] Nagayama M. Yano T. Atarashi K. Tanoue T. Sekiya M. Kobayashi Y.. (2020). TH1 cell-inducing *Escherichia coli* strain identified from the small intestinal mucosa of patients with Crohn's disease. Gut Microbes 12:1788898. doi: 10.1080/19490976.2020.1788898, PMID: 32691669 PMC7524366

[ref66] Narayana J. K. Aliberti S. Mac Aogáin M. Jaggi T. K. Ali N. Ivan F. X.. (2023). Microbial dysregulation of the gut-lung Axis in bronchiectasis. Am. J. Respir. Crit. Care Med. 207, 908–920. doi: 10.1164/rccm.202205-0893OC, PMID: 36288294 PMC10111978

[ref67] Ni Y. Qian L. Siliceo S. L. Long X. Nychas E. Liu Y.. (2023). Resistant starch decreases intrahepatic triglycerides in patients with NAFLD via gut microbiome alterations. Cell Metab. 35, 1530–1547.e8. doi: 10.1016/j.cmet.2023.08.002, PMID: 37673036

[ref68] Oh T. G. Kim S. M. Caussy C. Fu T. Guo J. Bassirian S.. (2020). A universal gut-microbiome-derived signature predicts cirrhosis. Cell Metab. 32, 878–888.e6. doi: 10.1016/j.cmet.2020.06.005, PMID: 32610095 PMC7822714

[ref69] Otten A. T. Bourgonje A. R. Peters V. Alizadeh B. Z. Dijkstra G. Harmsen H. J. M. (2021). Vitamin C supplementation in healthy individuals leads to shifts of bacterial populations in the gut-a pilot study. Antioxidants (Basel) 10:1278. doi: 10.3390/antiox10081278, PMID: 34439526 PMC8389205

[ref70] Ozen M. Piloquet H. Schaubeck M. (2023). *Limosilactobacillus fermentum* CECT5716: clinical potential of a probiotic strain isolated from human Milk. Nutrients 15:2207. doi: 10.3390/nu15092207, PMID: 37432320 PMC10181152

[ref71] Pan T. Zheng S. Zheng W. Shi C. Ning K. Zhang Q.. (2022). *Christensenella* regulated by Huang-Qi-Ling-Hua-san is a key factor by which to improve type 2 diabetes. Front. Microbiol. 13:1022403. doi: 10.3389/fmicb.2022.1022403, PMID: 36312936 PMC9597676

[ref72] Pascal V. Pozuelo M. Borruel N. Casellas F. Campos D. Santiago A.. (2017). A microbial signature for Crohn's disease. Gut 66, 813–822. doi: 10.1136/gutjnl-2016-313235, PMID: 28179361 PMC5531220

[ref73] Peterson C. T. Rodionov D. A. Osterman A. L. Peterson S. N. (2020). B vitamins and their role in immune regulation and Cancer. Nutrients 12:3380. doi: 10.3390/nu12113380, PMID: 33158037 PMC7693142

[ref74] Pittayanon R. Lau J. T. Leontiadis G. I. Tse F. Yuan Y. Surette M.. (2020). Differences in gut microbiota in patients with vs without inflammatory bowel diseases: a systematic review. Gastroenterology 158, 930–946.e1. doi: 10.1053/j.gastro.2019.11.294, PMID: 31812509

[ref75] Prochazkova P. Roubalova R. Dvorak J. Kreisinger J. Hill M. Tlaskalova-Hogenova H.. (2021). The intestinal microbiota and metabolites in patients with anorexia nervosa. Gut Microbes 13, 1–25. doi: 10.1080/19490976.2021.1902771, PMID: 33779487 PMC8018350

[ref76] Relizani K. Le Corf K. Kropp C. Martin-Rosique R. Kissi D. Déjean G.. (2022). Selection of a novel strain of *Christensenella minuta* as a future biotherapy for Crohn's disease. Sci. Rep. 12:6017. doi: 10.1038/s41598-022-10015-3, PMID: 35411016 PMC9001714

[ref77] Rooks M. G. Garrett W. S. (2016). Gut microbiota, metabolites and host immunity. Nat. Rev. Immunol. 16, 341–352. doi: 10.1038/nri.2016.42, PMID: 27231050 PMC5541232

[ref78] Ruaud A. Esquivel-Elizondo S. de la Cuesta-Zuluaga J. Waters J. L. Angenent L. T. Youngblut N. D.. (2020). Syntrophy via interspecies H_2_ transfer between *Christensenella* and *Methanobrevibacter* underlies their global Cooccurrence in the human gut. MBio 11:e03235-19. doi: 10.1128/mBio.03235-19 32019803 PMC7002349

[ref79] Sankaranarayanan R. Sekhon P. K. Ambat A. Nelson J. Jose D. Bhat G. J.. (2021). Screening of human gut bacterial culture collection identifies species that biotransform quercetin into metabolites with anticancer properties. Int. J. Mol. Sci. 22:7045. doi: 10.3390/ijms22137045, PMID: 34208885 PMC8269047

[ref80] Shannon P. Markiel A. Ozier O. Baliga N. S. Wang J. T. Ramage D.. (2003). Cytoscape: a software environment for integrated models of biomolecular interaction networks. Genome Res. 13, 2498–2504. doi: 10.1101/gr.1239303, PMID: 14597658 PMC403769

[ref81] Shetty S. Anegundi R. Shenoy P. A. Vishwanath S. (2023). Understanding antimicrobial susceptibility profile of *Finegoldia magna*: an insight to an untrodden path. Ann. Clin. Microbiol. Antimicrob. 22:30. doi: 10.1186/s12941-023-00583-1, PMID: 37098571 PMC10127037

[ref82] Shin J. Noh J. R. Choe D. Lee N. Song Y. Cho S.. (2021). Ageing and rejuvenation models reveal changes in key microbial communities associated with healthy ageing. Microbiome 9:240. doi: 10.1186/s40168-021-01189-5, PMID: 34906228 PMC8672520

[ref83] Sokol H. Pigneur B. Watterlot L. Lakhdari O. Bermúdez-Humarán L. G. Gratadoux J. J.. (2008). *Faecalibacterium prausnitzii* is an anti-inflammatory commensal bacterium identified by gut microbiota analysis of Crohn disease patients. Proc. Natl. Acad. Sci. USA 105, 16731–16736. doi: 10.1073/pnas.0804812105, PMID: 18936492 PMC2575488

[ref9001] Tamura K. Stecher G. Kumar S. (2021). MEGA11: Molecular Evolutionary Genetics Analysis Version 11. Mol. Biol. Evol. 38, 3022–3027. doi: 10.1093/molbev/msab120 33892491 PMC8233496

[ref84] Tavella T. Rampelli S. Guidarelli G. Bazzocchi A. Gasperini C. Pujos-Guillot E.. (2021). Elevated gut microbiome abundance of Christensenellaceae, Porphyromonadaceae and Rikenellaceae is associated with reduced visceral adipose tissue and healthier metabolic profile in Italian elderly. Gut Microbes 13, 1–19. doi: 10.1080/19490976.2021.1880221, PMID: 33557667 PMC7889099

[ref85] Tierney B. T. Yang Z. Luber J. M. Beaudin M. Wibowo M. C. Baek C.. (2019). The landscape of genetic content in the gut and Oral human microbiome. Cell Host Microbe 26, 283–295.e8. doi: 10.1016/j.chom.2019.07.008, PMID: 31415755 PMC6716383

[ref86] Upadhyaya B. McCormack L. Fardin-Kia A. R. Juenemann R. Nichenametla S. Clapper J.. (2016). Impact of dietary resistant starch type 4 on human gut microbiota and immunometabolic functions. Sci. Rep. 6:28797. doi: 10.1038/srep28797, PMID: 27356770 PMC4928084

[ref87] Uritskiy G. V. DiRuggiero J. Taylor J. (2018). MetaWRAP-a flexible pipeline for genome-resolved metagenomic data analysis. Microbiome 6:158. doi: 10.1186/s40168-018-0541-1, PMID: 30219103 PMC6138922

[ref88] Villanueva-Millan M. J. Leite G. Wang J. Morales W. Parodi G. Pimentel M. L.. (2022). Methanogens and hydrogen sulfide producing Bacteria guide distinct gut microbe profiles and irritable bowel syndrome subtypes. Am. J. Gastroenterol. 117, 2055–2066. doi: 10.14309/ajg.0000000000001997, PMID: 36114762 PMC9722381

[ref89] Wang S. Ma M. Liang Z. Zhu X. Yao H. Wang L.. (2022). Pathogenic investigations of *Streptococcus pasteurianus*, an underreported zoonotic pathogen, isolated from a diseased piglet with meningitis. Transbound. Emerg. Dis. 69, 2609–2620. doi: 10.1111/tbed.14413, PMID: 34871467

[ref90] Wang T. Sha L. Li Y. Zhu L. Wang Z. Li K.. (2020). Dietary α-linolenic acid-rich flaxseed oil exerts beneficial effects on polycystic ovary syndrome through sex steroid hormones-microbiota-inflammation Axis in rats. Front. Endocrinol. (Lausanne) 11:284. doi: 10.3389/fendo.2020.00284, PMID: 32670195 PMC7326049

[ref91] Waters J. L. Ley R. E. (2019). The human gut bacteria Christensenellaceae are widespread, heritable, and associated with health. BMC Biol. 17:83. doi: 10.1186/s12915-019-0699-4, PMID: 31660948 PMC6819567

[ref92] Wei B. Dalwadi H. Gordon L. K. Landers C. Bruckner D. Targan S. R.. (2001). Molecular cloning of a *Bacteroides caccae* TonB-linked outer membrane protein identified by an inflammatory bowel disease marker antibody. Infect. Immun. 69, 6044–6054. doi: 10.1128/iai.69.10.6044-6054.2001, PMID: 11553542 PMC98733

[ref93] Weiss A. S. Niedermeier L. S. von Strempel A. Burrichter A. G. Ring D. Meng C.. (2023). Nutritional and host environments determine community ecology and keystone species in a synthetic gut bacterial community. Nat. Commun. 14:4780. doi: 10.1038/s41467-023-40372-0, PMID: 37553336 PMC10409746

[ref94] Wu Y. W. Simmons B. A. Singer S. W. (2016). MaxBin 2.0: an automated binning algorithm to recover genomes from multiple metagenomic datasets. Bioinformatics 32, 605–607. doi: 10.1093/bioinformatics/btv638, PMID: 26515820

[ref95] Wu Y. Ye Z. Feng P. Li R. Chen X. Tian X.. (2021). *Limosilactobacillus fermentum* JL-3 isolated from "Jiangshui" ameliorates hyperuricemia by degrading uric acid. Gut Microbes 13, 1–18. doi: 10.1080/19490976.2021.1897211, PMID: 33764849 PMC8007157

[ref96] Xia L. C. Steele J. A. Cram J. A. Cardon Z. G. Simmons S. L. Vallino J. J.. (2011). Extended local similarity analysis (eLSA) of microbial community and other time series data with replicates. BMC Syst. Biol. 5:S15. doi: 10.1186/1752-0509-5-s2-s15, PMID: 22784572 PMC3287481

[ref97] Xiao H. Liu B. Yong J. Zhou H. (2019). Quantitative analysis and medium components optimizing for culturing a fastidious bacterium *Christensenella minuta*. bioRxiv. doi: 10.1101/632836

[ref98] Yadav U. C. Kalariya N. M. Srivastava S. K. Ramana K. V. (2010). Protective role of benfotiamine, a fat-soluble vitamin B1 analogue, in lipopolysaccharide-induced cytotoxic signals in murine macrophages. Free Radic. Biol. Med. 48, 1423–1434. doi: 10.1016/j.freeradbiomed.2010.02.031, PMID: 20219672 PMC2856750

[ref99] Yang Y. Gu H. Sun Q. Wang J. (2018). Effects of *Christensenella minuta* lipopolysaccharide on RAW 264.7 macrophages activation. Microb. Pathog. 125, 411–417. doi: 10.1016/j.micpath.2018.10.005, PMID: 30290268

[ref100] Yang Y. Wu C. (2022). Targeting gut microbial bile salt hydrolase (BSH) by diet supplements: new insights into dietary modulation of human health. Food Funct. 13, 7409–7422. doi: 10.1039/D2FO01252A, PMID: 35766281

[ref101] Yoon S. H. Ha S. M. Kwon S. Lim J. Kim Y. Seo H.. (2017). Introducing EzBioCloud: a taxonomically united database of 16S rRNA gene sequences and whole-genome assemblies. Int. J. Syst. Evol. Microbiol. 67, 1613–1617. doi: 10.1099/ijsem.0.001755, PMID: 28005526 PMC5563544

[ref102] Zafar H. Saier M. H. Jr. (2021). Gut *Bacteroides* species in health and disease. Gut Microbes 13, 1–20. doi: 10.1080/19490976.2020.1848158, PMID: 33535896 PMC7872030

[ref103] Zamani S. Taslimi R. Sarabi A. Jasemi S. Sechi L. A. Feizabadi M. M. (2019). Enterotoxigenic *Bacteroides fragilis*: a possible etiological candidate for bacterially-induced colorectal precancerous and cancerous lesions. Front. Cell. Infect. Microbiol. 9:449. doi: 10.3389/fcimb.2019.00449, PMID: 32010637 PMC6978650

[ref104] Zhai L. Huang C. Ning Z. Zhang Y. Zhuang M. Yang W.. (2023). *Ruminococcus gnavus* plays a pathogenic role in diarrhea-predominant irritable bowel syndrome by increasing serotonin biosynthesis. Cell Host Microbe 31, 33–44.e5. doi: 10.1016/j.chom.2022.11.006, PMID: 36495868

[ref105] Zhang Y. Thompson K. N. Branck T. Yan Y. Nguyen L. H. Franzosa E. A.. (2021). Metatranscriptomics for the human microbiome and microbial community functional profiling. Annu. Rev. Biomed. Data Sci. 4, 279–311. doi: 10.1146/annurev-biodatasci-031121-103035 34465175

[ref106] Zhang Q. Zhao Q. Li T. Lu L. Wang F. Zhang H.. (2023). *Lactobacillus plantarum*-derived indole-3-lactic acid ameliorates colorectal tumorigenesis via epigenetic regulation of CD8^+^ T cell immunity. Cell Metab. 35, 943–960.e9. doi: 10.1016/j.cmet.2023.04.015, PMID: 37192617

