# Supplementary material for: Christensenella minuta interacts with multiple gut bacteria
Source: Front Microbiol. 2024 Feb 19;15:1301073. doi: 10.3389/fmicb.2024.1301073 (PMC10910051; doi:10.3389/fmicb.2024.1301073)
Supplement: Supplementary file 2 [file Image_1.PDF]

Supplementary Material

1.1 Supplementary Figures

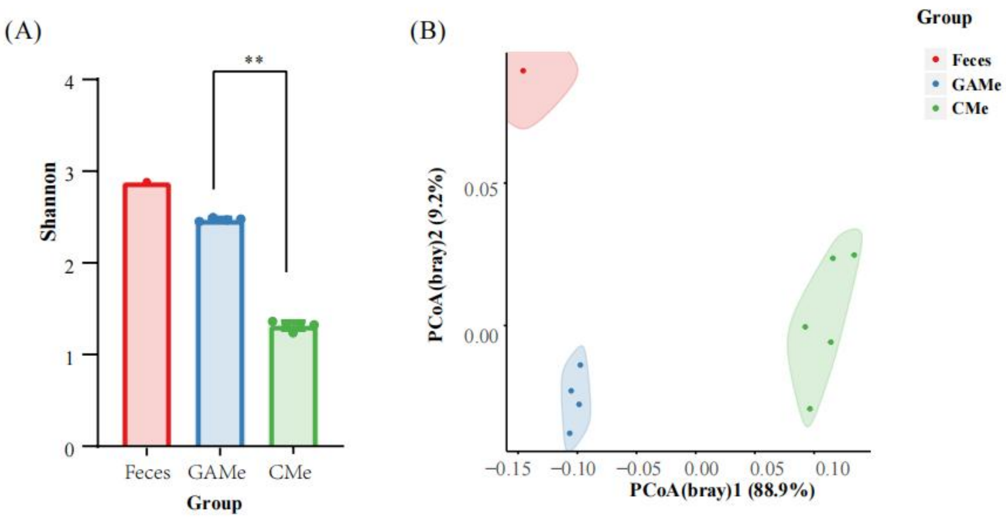

Supplementary Figure 1. Impacts of *C. minuta* on the diversity of fecal microbiomes composition. **(A)** Alpha diversity of Feces, GAMe, and CMe microbial communities. The alpha diversity, measured by the observed Shannon index, was plotted. The mean and standard error of the mean (SEM) were shown as lines connecting the mean values of each group, and indicated by error bars. Sample values were represented as dots. **(B)** The principal co-ordinates analysis (PCoA) analysis based on Bray-Curtis distance was used to study the community composition of three groups. Each dot represents one sample.
